# Climate change and human health in Vietnam: a systematic review and additional analyses on current impacts, future risk, and adaptation

**DOI:** 10.1016/j.lanwpc.2023.100943

**Published:** 2023-11-15

**Authors:** Nu Quy Linh Tran, Huynh Thi Cam Hong Le, Cong Tuan Pham, Xuan Huong Nguyen, Ngoc Dang Tran, Tuyet-Hanh Thi Tran, Son Nghiem, Thi Mai Ly Luong, Vinh Bui, Thong Nguyen-Huy, Van Quang Doan, Kim Anh Dang, Thi Hoai Thuong Do, Hieu Kim Thi Ngo, Truong Vien Nguyen, Ngoc Huy Nguyen, Manh Cuong Do, Tuan Nghia Ton, Thi Anh Thu Dang, Kien Nguyen, Xuan Bach Tran, Phong Thai, Dung Phung

**Affiliations:** a Centre for Environment and Population Health, School of Medicine and Dentistry, Griffith University, Australia; b Child Health Research Centre, Faculty of Medicine, University of Queensland, Australia; c FHI 360 Vietnam Office, Hanoi, Vietnam; d Centre for Scientific Research and International Collaboration, Phan Chau Trinh University, Quang Nam, Vietnam; e University of Medicine and Pharmacy at Ho Chi Minh City, Ho Chi Minh City, Vietnam; f Hanoi University of Public Health, Hanoi, Vietnam; g Department of Health Economics, Wellbeing and Society, Australian National University, Australia; h Faculty of Environmental Sciences, Vietnam University of Science, Hanoi, Vietnam; i Faculty of Science and Engineering, Southern Cross University, Australia; j Centre for Applied Climate Sciences, University of Southern Queensland, Australia; k Centre for Computational Sciences, University of Tsukuba, Japan; l Queensland Alliance for Environmental Health Sciences, The University of Queensland, Australia; m Pham Ngoc Thach University of Medicine, Vietnam; n Vietnam National University - Vietnam Japan University, Hanoi, Vietnam; o Health Environment Management Agency, Ministry of Health, Vietnam; p Office of WHO Representative in Vietnam, Vietnam; q Hue University of Medicine and Pharmacy, Hue University, Hue City, Vietnam; r Hue University of Economics, Hue University, Hue City, Vietnam; s Hanoi Medical University, Vietnam; t School of Public Health, The University of Queensland, Australia

**Keywords:** Climate change, Health impacts, Adaptation, Vietnam, Systematic review

## Abstract

This study aims to investigate climate change's impact on health and adaptation in Vietnam through a systematic review and additional analyses of heat exposure, heat vulnerability, awareness and engagement, and projected health costs.

Out of 127 reviewed studies, findings indicated the wider spread of infectious diseases, and increased mortality and hospitalisation risks associated with extreme heat, droughts, and floods. However, there are few studies addressing health cost, awareness, engagement, adaptation, and policy.

Additional analyses showed rising heatwave exposure across Vietnam and global above-average vulnerability to heat. By 2050, climate change is projected to cost up to USD1-3B in healthcare costs, USD3-20B in premature deaths, and USD6-23B in work loss.

Despite increased media focus on climate and health, a gap between public and government publications highlighted the need for more governmental engagement. Vietnam's climate policies have faced implementation challenges, including top-down approaches, lack of cooperation, low adaptive capacity, and limited resources.

**Research in context**

**Evidence before this study**

Although the number of climate-health studies has significantly increased in Vietnam since 2012, these studies have not been properly reviewed to inform the health sector's action plans, strategies, and policies on climate change and health. A search of peer-reviewed and preprint systematic, scoping, or rapid review of literature without language restrictions in PubMed and EMBASE using the search terms including “Vietnam”, “climate change”, “extreme weather”, “health”, “health sectors”, “policy”, and “adaptation” and found three reviews conducted in Vietnam had concentrated on the relationship between climatic factors and specific health outcomes. These studies included a scoping review of climate-related disasters and health impacts, a review of climate change and water-related diseases in the Mekong Delta Region in 2015, and an updated review in 2023. These reviews did not consider a wide range of climatic factors and their effects on human health, nor did they provide evidence on health-related costs, future projection, and the health vulnerability and adaptability of the population to climate change across Vietnam.

**Added values of this study**

This study is the most comprehensively systematic review of climate change and human health in Vietnam to date. It synthesised 127 existing studies on various aspects including the effects of different climate factors on a wide range of health outcomes, climate change awareness and engagement, adaptation strategies, and policy implementation. Moreover, the study conducted four additional analyses focusing on heat exposure, heat vulnerability, public awareness and engagement, and the estimated health costs associated with climate change. The combination of a systematic review and findings from the additional analyses has enabled a comprehensive understanding of the climate change-health relationship in Vietnam. By identifying the research gaps present in the existing literature and the current state of policy implementation in Vietnam, this review has given valuable recommendations for the research agenda and adaptation policy in Vietnam.

**Implications of all the available evidence**

Vietnam, a low-middle-income nation in Southeast Asia, is one of the most vulnerable countries to the impacts of climate change. The results of this comprehensive review can help stakeholders, policymakers, and researchers develop evidence-based interventions, strategies and policies that support resilience, safeguard populations at risk, and guarantee the long-term sustainability of health systems in the face of environmental change. Future research is needed to support policymakers in prioritising financial and human resources to address the appropriate health risks of climate change, vulnerable populations, and adaptation strategies.

## Introduction

Climate change exerts substantial and escalating impacts on human health, with projections indicating a further intensification throughout the 21st century.^1^ The health impacts can be directly due to extreme weather events such as heatwaves,^2^^,^^3^ floods, and the intensity of storms^4^^,^^5^ or indirectly due to the consequent changes in environmental conditions, which lead to an increase in waterborne diseases,^6^ foodborne diseases,^7^^,^^8^ vector-borne diseases,^9^^,^^10^ nutritional insecurity,^11^ and mental health disorders.^4^^,^^5^ However, health vulnerability to climate change is not evenly distributed but varies by geographical settings due to the differences in environmental and socioeconomic conditions as well as adaptive capacity.^12^, ^13^, ^14^

Vietnam, a low-middle-income country in Southeast Asia, is one of the tropical countries most vulnerable to the risk of climate change impacts.^15^ Vietnam is frequently exposed to hydro-meteorological hazards comprising severe storms, cyclones, typhoons, floods, and landslides. Approximately 70% of residents live in coastal communities with high exposure to storms and floods, which have been intensifying due to climate change.^16^ Each year, floods affect an estimated 930,000 people with a Gross Domestic Product (GDP) loss of USD2.6 billion.^17^ A further estimation by the World Bank and Asian Development Bank indicates that 3 to 9 million people would be affected by fluvial floods in 2035–2044 and 6 to 12 million people would be affected by coastal floods in 2070–2100.^18^ Most of the extensive low-lying coastline and low-lying delta regions in Vietnam are highly vulnerable to rising sea levels.^16^ In recent years, droughts have occurred in most regions of the country successively. The most severe drought in 90 years occurred in 2016, impacting over 275,260 hectares of rice paddies (constituting approximately 4% of the total rice paddy area) and nearly 189,880 hectares of perennial crops across 18 provinces.^19^ In 2019–2020, severe droughts and saltwater intrusion were observed in 10 provinces of the Mekong Delta Region (MDR), resulting in limited water access for over 200,000 households and disrupted basic services for around 685,000 people.^20^ The World Bank also anticipates a 3.5% reduction in national income by 2025 due to climate change-related hazards.^21^

In 2018, the Vietnam Ministry of Health (MOH) adopted an action plan for adaptation to climate change in the health sector for 2019–2030 with the vision of 2050.^22^ A recent review of the progress of the implementation of the action plan in 2022 stated that promoting scientific research on the effects of climate change on health and the health sector's adaptive solutions should be strongly prioritised.^23^ Climate-health evidence plays a crucial role in informing effective, equitable, and timely adaptation responses and strategies for health sectors. Despite a significant increase in the number of climate-health studies observed in Vietnam since 2012, the studies have not been systematically reviewed or evaluated to inform climate change and health action plans, strategies, and policies in the health sector. The objectives of this review are i) to provide a comprehensive synthesis of evidence on the impacts of climate change on human health; ii) to promote climate-health research to inform the implementation of adaptation policies; iii) to contribute to the knowledge-sharing communities of practice in climate change and health in Vietnam. To meet these three objectives, the review will address the following specific research questions:

1. What are the trends and characteristics of the studies on climate change and health already conducted?
2. What is the current evidence on climate-related health risks, exposures, and vulnerability?
3. What are the projections for climate-related health risks in the future?
4. What is the level of public and political awareness and engagement in climate change and health?
5. How effective is climate-health adaptation policy in Vietnam, for whom, under what conditions, and how effective is the implementation?

## Methods

We conducted a systematic review and four additional analyses to answer Research Questions one to five.

### Systematic review

#### Search strategy

We searched scientific articles in both English and Vietnamese up to 5 December 2022. The scientific articles were collected from PubMed, Web of Science, Embase, and CINAHL for articles in English, and Google Scholar and the websites of domestic journals for articles in Vietnamese. We combined three categories of search terms: country of Vietnam, climate change, and health. The full list of search terms is presented in Supplement #2. Search terms on specific mediators of the health outcomes of climate change, such as food insecurity, were not included in the review. We identified additional relevant literature from a manual search of references in the included articles.

#### Selection criteria and screening

We included articles that described: i) the health impacts of climate change-related factors (CCRFs) on health and related economic burdens; ii) the health vulnerability to CCRFs; iii) the projected impacts of CCRFs and related economic burdens in the future; iv) public awareness of, and engagement in, climate change and health; and v) climate change adaptation interventions in the health sector. The CCRFs include seasonality, variation of weather parameters (e.g., temperatures, humidity, and precipitation), and extreme weather events (e.g., heat waves, extreme colds, floods, heavy rainfalls, droughts, and storms). Health is defined to include physical, mental, emotional, and social health and wellness. We excluded articles which meet the exclusion criteria, namely a) indoor work environments, non-climate hazards due to geologic events (e.g., earthquakes), and non-anthropogenic climate change (e.g., due to volcanic eruptions); b) chamber studies looking at the effects of controlled weather factors on health; c) climatic and/or meteorological variables independently of health outcomes; d) books, book chapters, theses, articles in the media; e) commentaries, letters to the editor, conference abstracts/proceedings, perspectives/viewpoints, primers, protocol/frameworks, replies from authors, opinion pieces; g) studies whose full texts were unavailable; and h) studies on non-human subjects.

Articles were screened in two stages using Covidence software.^24^ The titles and abstracts were screened by KAD, HTCHL, VTN, THTD, HKTN, NQLT, and CTP, and then full-text reviews were performed by KAD, HTCHL, XHN, NQLT, TMLL, and CTP. Articles that were voted “yes” by two voters were then included in the next stage. Any conflicts were resolved via discussion between two yes-voters. A third relevant reviewer was assigned to review and make the final decision for the unresolved articles. “Unsure” articles were reviewed by the lead reviewer (DP) for the final decision. The Covidence platforms for English papers and Vietnamese papers were kept separate.

#### Data extraction and analysis

Data were extracted using Microsoft Excel by one reviewer and supplemented by another one. The data focused on citation information, study characteristics, and the key findings of research topic domains comprising climate-related health impacts, vulnerability, future risk, health-economic loss, and awareness and engagement. The data also included limitations and recommendations provided by the authors and reviewers. The list of all included studies and data extraction is provided in Supplement #3.

A PRISMA flowchart was created to demonstrate the article selection process and reasons for exclusion. This also ensures the replicability and transparency of the process. Descriptive analysis was used to examine the trend and distribution of studies, which were then visualised by graphs and maps. A narrative synthesis approach was applied to qualitatively analyse data.^25^ We first developed a preliminary synthesis by grouping studies by topic domains, overviewing the characteristics of included studies, and describing the patterns across the studies. Then we explored the relationship in the data by looking at the consistency and heterogeneity of the findings across the studies and investigating the potential factors that might explain any differences in the direction of findings. Finally, we evaluated the robustness of the synthesis product by analysing the strengths and limitations of the evidence. Quality appraisal of studies included in the systematic review was conducted using a framework based on the Mixed Methods Appraisal Tool (MMAT), which enabled appraisal of evidence in reviews that contain qualitative, quantitative, and mixed methods studies.^26^ One reviewer appraised the included article, and another reviewer checked the validity of the appraisal. The two reviewers then met to discuss judgments as needed.

### Additional analysis and policy review

We conducted four additional analyses using the existing data obtained from published data sources to supplement the findings of the systematic review and address the relevant research questions.

We tracked down the progress of two heat-health indicators: the Heat Exposure Vulnerability Index (HEVI) and Exposure of Vulnerable Population to Heatwaves (EVPH). We referred to the methods provided by the *Lancet Countdown 2021* and the *Australian Countdown*.^27^^,^^28^ Additionally, due to the scarcity and heterogeneity of reviewed studies that estimated the cost of climate change and extreme heat on health in Vietnam, we estimated the costs of climate change on human health using a “back-of-the-envelope” approach.^29^ The details of the methods are provided in Supplement #4.

We explored the political engagement in, and public awareness of, climate change and health by analysing media coverage of climate change and health in both governmental and public media. We used several combinations of keywords for climate change and health in the search, which was conducted using a web crawler. We only selected media publications that contained all search keywords to avoid including media publications that were not relevant to the impact of climate change on health. The specific keywords are provided in Supplement #5.

Regarding the policy review, firstly, we searched information from the official legal databases of Vietnam,^30^ namely the websites of the MOH, General Statistics Office, and Ministry of Natural Resources and Environment to compile a comprehensive list of national policies related to climate change adaptation. The list then was double-checked by experts in policy, climate change, and the health sector (Supplement #6). Secondly, the experts discussed and summarised priority strategies and action plans in climate change-related health issues to identify challenges and barriers that hinder the implementation of these policies. Lastly, the authors propose recommendations that can enhance the implementation of climate change and health adaptation policies in the health sector.

### Role of the funding source

This work received no funding.

## Results

### The trend and characteristics of climate change and health studies

**Fig. 1:**
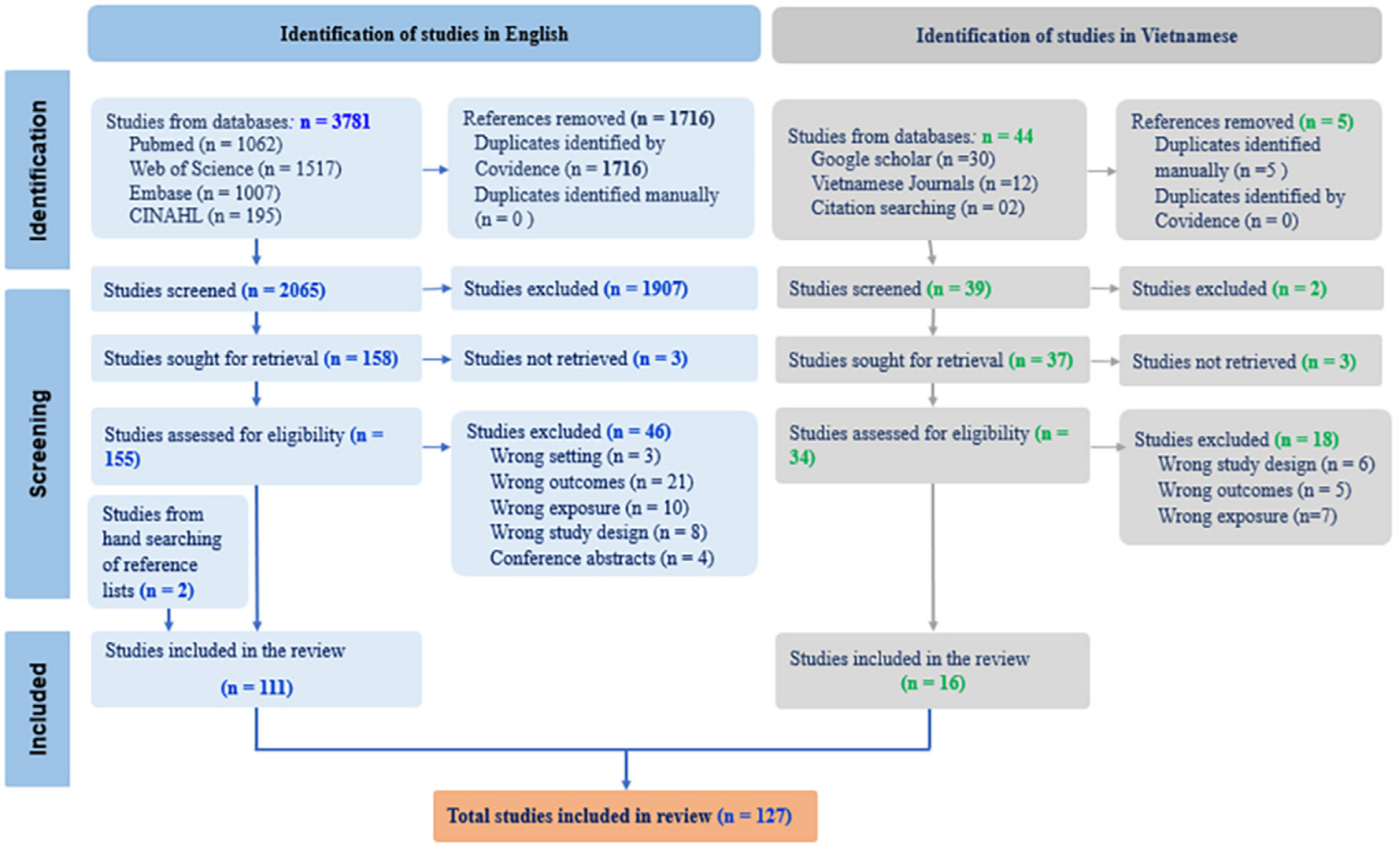
Flowchart of assessment of eligible articles.

Database searches identified 3781 articles in English and 44 articles in Vietnamese. After removing 1721 duplicates (English and Vietnamese articles), we screened 2104 articles, with 1907 English and 02 Vietnamese articles excluded at the title and abstract screening stage because they were not eligible. This left 189 articles to assess their eligibility for full-text screening. Ultimately, we identified 127 articles (111 in English and 16 in Vietnamese) that fulfilled the inclusion criteria for the systematic review (Fig. 1).

**Fig. 2:**
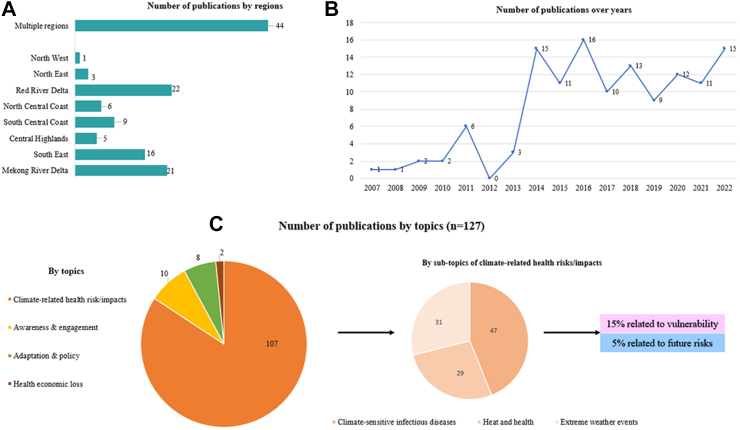
Distribution of studies on climate change and health in Vietnam by regions, years, and topics (n = 127).

Fig. 2A presents the distribution of studies concerning the study location. One-third (44/127) of studies were conducted in multiple regions while the remaining studies primarily concentrated on areas within a specific ecological region. The Red River and MDR regions were the most studied. These regions also have the largest population and serve as significant economic centres in Vietnam. Fig. 2B presents the distribution of studies by years. There is an increasing trend in published research over the years, starting from 2007, with a significant increase starting from 2014.

Most studies focused on climate-related health risks/impacts while a small proportion of studies looked at awareness, engagement, adaptation, and policy (Fig. 2C). Within the articles on climate-related health risks/impacts (n = 107), nearly half focused on climate-sensitive infectious diseases (n = 47), while a quarter discussed heat and health (n = 29), and extreme weather events (n = 31). Out of the reviewed articles, only 15% were related to the topic of vulnerability, and 5% were related to future risks.

### Climate change impacts, exposure, and vulnerability

#### Impacts of temperature on health

Most studies examined the effects of high and/or low temperatures on mortality and hospitalisation risks while only 5 studies included a projection of heat-related health hazards and spatial heat-health vulnerability. Time series was the most common study design applied to examine the effects of temperatures. A j-shape relationship between temperatures and all-cause mortality was observed in two single cities,^31^^,^^32^ in which high temperatures with a short lag (0–3 days) increased the risk of all-cause mortality from a threshold of approximately 29 °C. In contrast to temperature-mortality, most studies^33^, ^34^, ^35^, ^36^ of the temperature-hospitalisation relationship in multiple provinces/cities examined the linear but not the non-linear effects of temperatures. For a 1 °C increase in average temperature, the risk of all-cause hospitalisations increased by 1.1%–1.3%.^34^^,^^35^ Heatwaves significantly increased the risk of all-cause hospitalisations, and the effect size was greater in the North than in the South (5.4% vs. 1.3%).^37^

For the cause-specific hospitalisations, high and low temperatures were associated with elevated risk of respiratory diseases^34^^,^^35^^,^^38^ and cardiovascular diseases,^34^^,^^39^, ^40^, ^41^ while high temperatures were linked to risk of infectious diseases,^33^, ^34^, ^35^^,^^42^^,^^43^ kidney diseases,^44^ and mental health disorders.^45^, ^46^, ^47^ The vulnerable groups to heat-related health risks included older people,^31^^,^^32^^,^^35^^,^^39^^,^^48^ children,^42^^,^^49^, ^50^, ^51^ and those frequently working outdoors such as farmers and traffic police.^52^, ^53^, ^54^ The heat-health relationships were modified by the population density, poverty rate, illiteracy rate, household income, access to water supply and hygienic toilets, and the proportion of preschool children and women.^33^^,^^35^ A vulnerability assessment in multiple provinces revealed that Southern provinces were more vulnerable to the health impact of heatwaves than the North.^55^ It has been projected that the heat index will increase from 0.0777 °C to 0.080 °C/year corresponding to the Representative Concentration Pathway (RCP) scenarios RCP4-5 and RCP8-5,^56^ the net excess heat-related mortality rates could rise from 3% to 26% corresponding to the RCP2-6, RCP4-5, RCP6-0, and RCP8-5 scenarios by the end of this century (2090–2099) for Vietnam.^57^ Meanwhile, two studies anticipated that excess mortality would increase particularly in tropical countries such as Vietnam (10.34% per 1 °C increase)^58^ and the number of hospital admissions would increase by an additional 10,000 cases in the most affected province in the MDR in 2100.^59^

It is challenging to evaluate the consistency and heterogeneity of the effect sizes of high temperatures on health due to the small number of studies that have consistent measurements of exposures and outcomes. For example, the studies used different heatwave definitions (≥90th percentile for ≥3 days consecutively^37^ or ≥97th percentile for ≥2 consecutive days).^31^ In addition, there was inconsistency in characterising the heat wave effects, such as comparing hospitalisations between heatwave and non-heatwave periods^45^; or examining the main effect (temperature intensity) and added effect (duration of heatwaves).^46^ Some studies used disease groups,^33^^,^^40^ whereas others used one specific disease as the health outcome.^41^ More than half of the studies were ranked with low scores or could not be assessed for quality (19 studies with a score of three or less or ‘can't tell’). These studies might have encountered biases in outcome and exposure measurements, such as the absence of International Classification of Diseases codes for cause-specific mortality data and temperature data collected from a single station, potentially lacking representativeness for broader geographical areas.^33^^,^^35^

**Fig. 3:**
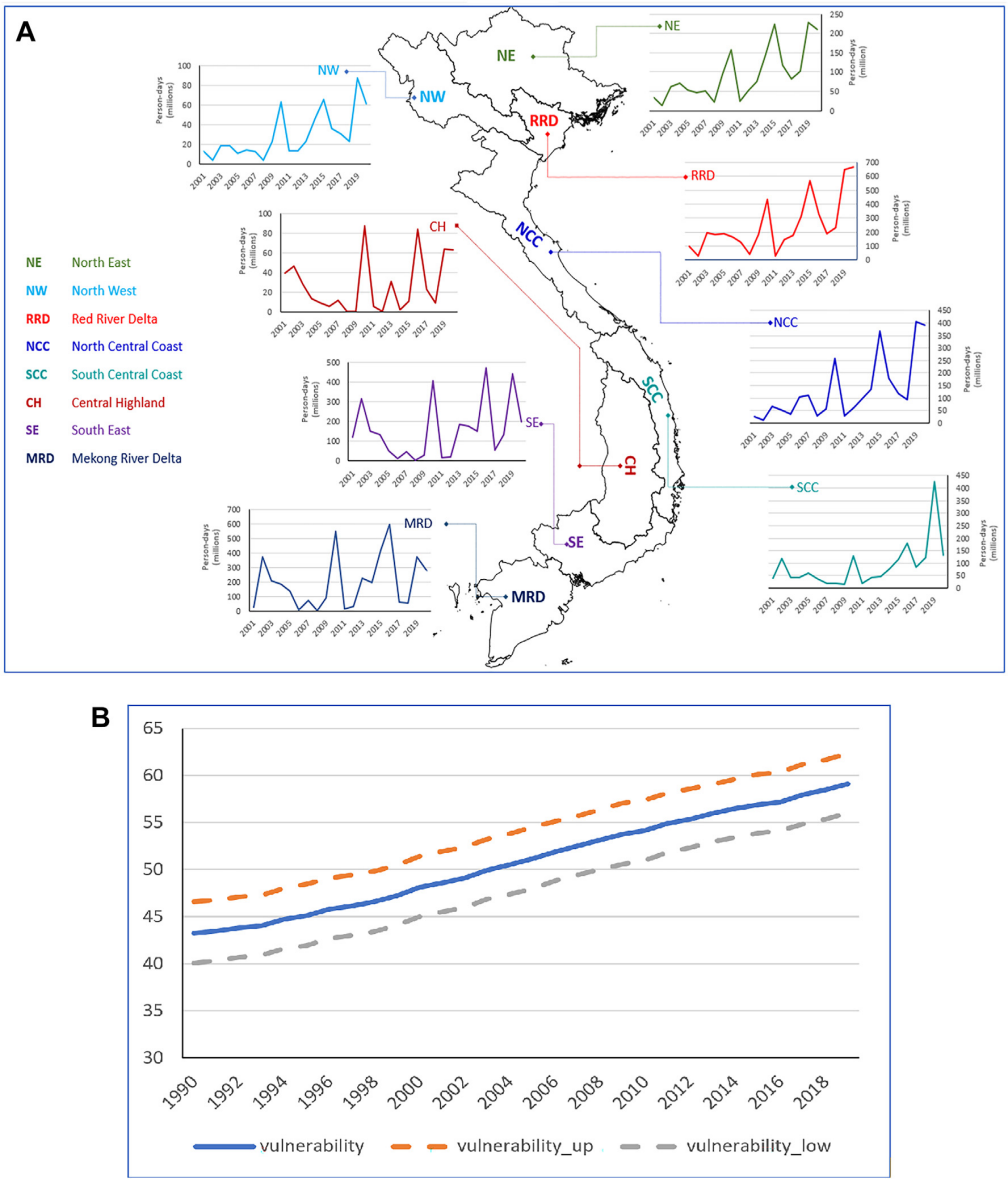
A–Exposure of populations to heatwaves (person-days) across 8 ecological regions of Vietnam, 2001–2020. B–Heat exposure vulnerability index for the Vietnamese population, 1990–2018.

The findings of additional analyses of heatwave exposures and heat exposure vulnerability are shown in Fig. 3. Results show a steady increase in person-days of exposure in all regions, with an annual average of 20 million additional person-days of heatwave exposures in each region during the study period. The highest increase was observed in the Red River Delta (approximately 29 million person-days per year), and the lowest one was observed in the North-West region (approximately 9 million person-days per year). Compared to the first year of the 21st century (2001), across the country, there were 200 million additional person-days of heatwave exposures across the country in 2020 (Fig. 3A). The heat exposure vulnerability index (HEVI) for the Vietnamese population combines factors such as the proportion of the population over 65, the prevalence of respiratory diseases, cardiovascular diseases, and diabetes among that group, and the urban population proportion. The HEVI for Vietnam from 1990 to 2020 reveals an increasing trend which is far higher than the global average and the average for all World Health Organization (WHO) regions (Fig. 3B).^27^ For example, in 2017, the HEVI for Vietnam stood at 57, whereas the global average was 36. The index is scaled from zero to 100, with higher values indicating increased vulnerability.

#### Impacts of extreme weather events

Among 31 studies about health issues linked to extreme weather events, 20 addressed the effects on health, nine evaluated vulnerabilities, and two identified future risks. Among Southeast Asian nations, Vietnam experienced the most frequent floods, especially tropical cyclone flooding,^60^^,^^61^ responsible for most disaster-related fatalities, followed by tropical cyclones and flash floods.^62^ Floods are predicted to increase in severity over the next decades, along with the affected populations.^63^^,^^64^ The most reported health impacts of floods were the increase in deaths and injuries,^62^^,^^65^^,^^66^ water-transmitted diseases,^67^ pink eyes (conjunctivitis), dermatitis,^68^ and hospitalisation risk.^69^^,^^70^ For example, the 2011 extreme annual flood in the MDR increased non-external cause hospitalisations, infectious diseases, and respiratory diseases by 7.2%, 16.4%, and 25.5%, respectively.^70^ Also, high river water levels in the MDR significantly increased the risk of pediatric hospital admissions, especially among the under-five age group.^69^ The lag effect of floods can be observed up to 15 days,^69^ and the risk of hospitalisation increases with the severity of the flood.^70^ In terms of extreme rainfall, all five existing studies consistently reported negative impacts on children, including being underweight,^71^ low-birth weight,^72^ lower height-for-age index,^73^ and lower overall health status.^74^ Children in rural areas and ethnic minorities were significantly affected by floods and excessive rainfalls.^69^^,^^73^^,^^74^ However, a slightly above-average rainfall during gestation was positively associated with better cognitive development.^75^ This may be because increased rainfall could lead to enhanced availability of fresh food, cleaner air, and reduced environmental pollutants.

The negative health impacts of droughts reported included increased risk of hospitalisation,^70^ reduced overall health conditions, and increases in health expenditures,^76^ as well as decreased body mass index and height-for-age among children.^77^ A time-series study in two MDR provinces found that river drought increased the risk of all-cause, respiratory, and renal hospitalisations by 2%, 2%, and 7%, respectively, with an estimated additional cost during the 1995–2014 period of over USD360,300.^78^ Another study reported that sudden health deterioration related to droughts resulted in an increase in health expenditures ranging from 9% to 17% of total consumption.^76^ Other studies reported an increase in self-reported mental health problems following typhoons,^79^^,^^80^ an increase in hypertension hospitalisation risk in areas affected by salinity intrusion,^81^ and higher numbers of injuries, traffic accidents, and deaths during storms and damaging cold days.^65^ In terms of vulnerability assessment, models at both global and regional scales ranked Vietnam as among the countries most affected by multiple climatic hazards.^82^^,^^83^ They also found that each ecological region was characterised by different disasters.^67^^,^^84^ For example, the North Central and South Central Coasts were at a high risk of floods and tropical depressions while the Red River and MDR were mostly affected by drought and saltwater intrusions.^84^ However, the methods, frameworks, and indicators of vulnerability assessments varied across studies. Also, these assessments covered different and multiple aspects of vulnerability including health, livelihood, and social vulnerability.^84^, ^85^, ^86^, ^87^, ^88^, ^89^, ^90^

In summary, among 20 studies on health-related to extreme weather events, the adverse impacts of floods on hospitalisation risks and of extreme rainfall on children's health had a high level of consistency among studies. In contrast, the impacts of droughts, tropical cyclones, and salinity were only mentioned in a limited number of studies which had different health outcomes; therefore, we cannot assess the issues of heterogeneity or consistency.^91^ Seven out of 20 studies on health impacts ranked high scores for quality with cohort or longitudinal study design, using large and reliable data for quantitative assessment of the effects of floods, rainfall, droughts, and salinity on the burden of disease including hospitalisation risk and child health indicators.^69^, ^70^, ^71^, ^72^^,^^75^^,^^80^^,^^81^ Other studies had lower scores of quality (with 3/5 or lower or ‘can't tell’) and most of them relied on self-reported health measures, rapid post-disaster damage estimation, and indirect estimations. These studies have provided preliminary evidence of disaster-related health consequences; however, it is difficult to generalise the results or determine vulnerable populations, contributing factors, and long-term health impacts.

#### Climate-sensitive infectious diseases

Evidence regarding climate-sensitive infectious diseases in Vietnam mostly focuses on four groups: mosquito-borne diseases; waterborne diseases; respiratory infectious diseases; and other modes of transmission. Only three studies analysed the vulnerability to dengue^92^; waterborne diseases,^93^ and infectious diseases in general.^94^ Among the four infectious disease groups, mosquito-borne diseases received the most attention with 18 studies on dengue^95^, ^96^, ^97^, ^98^, ^99^, ^100^, ^101^, ^102^, ^103^, ^104^, ^105^, ^106^, ^107^, ^108^, ^109^, ^110^, ^111^, ^112^ and five studies on malaria.^110^^,^^111^^,^^113^, ^114^, ^115^

Evidence of increased temperature with a higher incidence of dengue was consistent across all studies^95^, ^96^, ^97^, ^98^, ^99^, ^100^, ^101^, ^102^, ^103^, ^104^, ^105^, ^106^, ^107^, ^108^, ^109^, ^110^; e.g., 1 °C increase in temperature results in a 5% (3–7.4%) increase in dengue, compared to a 0.4%–2.5% increase in other infectious diseases.^110^ Dengue-humidity positive associations were found in 6 out of 8 studies^95^^,^^97^^,^^99^^,^^102^^,^^107^^,^^108^ and negative associations in the rest.^101^^,^^110^ Ten studies showed a positive association between rainfall and dengue, but one reported that this pattern can reverse when rainfall exceeds 550 mm per month.^103^ One-month-lag-effects were suggested to develop prediction models for dengue^92^^,^^109^^,^^116^; and the authors suggested that such models should also consider non-climatic factors (entomological, virological, and anthropological factors).^116^

Another mosquito-borne disease—malaria—has significantly decreased, and by 2017 more than 40 of 63 provinces were malaria-free. However, the disease is still endemic in some mountainous and rural areas in the Central Highlands, South Central Coast, and the MDR.^111^^,^^113^ The impact of temperature and rainfall on malaria varied. Heterogeneity across studies due to differences in ecological zones and seasonal patterns,^113^, ^114^, ^115^ and the temperature-malaria association was much lower (0.4%) than that of dengue (5%).^110^ Therefore, the authors recommended that further studies on malaria should be carried out at a regional level and should consider other contributing factors including forest cover and poverty.^113^

Waterborne diseases including diarrhoea and intestinal diseases were analysed in nine studies. These showed positive associations between waterborne diseases and temperatures (2–4 weeks prior), humidity, and rainfalls (at lag 4–6 days) in the MRD^117^, ^118^, ^119^; or negative associations in Ho Chi Minh City.^120^ However, the climate-water-borne disease relationship varied (e.g., 0.5%–2.5% change with a 1 °C increase in temperature, 0.3%–1.7% change with 1% increase of humidity^110^) or was even not significant. The authors suggest that water quality should also be considered as a co-factor with climatic factors.^111^^,^^121^

Respiratory infectious diseases including influenza/influenza-like, mumps, chickenpox, and tuberculosis were investigated in nine original studies^38^^,^^110^^,^^117^^,^^122^, ^123^, ^124^, ^125^, ^126^, ^127^ and one review.^94^ These illustrated contradictory impacts of climatic factors on respiratory infectious diseases in different research settings. In the North with hot and cold seasons; the highest hospitalisation risk for respiratory infectious diseases was observed at 13 °C (RR = 1.39) in cold weather and 33 °C (RR = 1.21) in hot weather, with colder temperatures having more significant impacts.^38^ Conversely, in the South with consistently warm weather, respiratory infectious diseases showed a negative association with temperature (IRR ranged from 0.85 to 0.92) but a positive association with humidity^123^^,^^127^ and dew point (IRR ranged from 1.08 to 1.26).^122^ Among infectious diseases with other modes of transmission, hand-foot-mouth disease was the most studied. These studies found consistently positive associations with temperatures and humidity,^43^^,^^110^^,^^128^, ^129^, ^130^, ^131^, ^132^ except for a study in the Central Highland that reported a negative association^133^; however longer lag-day reduced the magnitude of associations (e.g., 1 °C increase in temperature results in 1.7% and 0.3% increases of hand-foot-mouth disease at lag 0 and lag 1 day, respectively).^132^ Meanwhile, rainfall showed both positive and negative associations with hand-foot-mouth disease in different research locations.^43^^,^^110^^,^^128^^,^^130^^,^^131^ The heterogeneity of the effects of rainfalls on hand-foot-mouth disease was higher than that of temperatures or humidity.^132^ Other IDs such as children's nervous system infection,^134^ plague,^135^ AIDS-associated *Penicillium marneffei* infection,^136^ or rabies^130^ were only mentioned in a few studies with insufficient information on climate-infectious disease association.

Out of 49 studies, only three analysed the vulnerability to dengue^92^; waterborne diseases,^93^ or infectious diseases in general.^94^ The sensitive population included children^92^ and families with young children.^3^ The remaining study^94^ only mentioned children, people living in coastal areas, and those impacted by natural disasters without specifically analysing them. Two studies introduced and evaluated the accuracy of dengue forecasting models, using Seasonal Autoregressive Integrated Moving Average models^116^ and deep learning models^137^ to predict dengue in the short term, and recommended incorporating climatic and non-climate factors into predictive models for climate-sensitive diseases. However, long-term future risks of IDs were not predicted.

Despite the large number of studies on climate change and infectious diseases, the quality and consistency of studies were questionable. One-third of the original studies on climate impacts on infectious diseases ranked high scores for quality. They quantitatively measured the effects of changes in temperature, precipitation, humidity, and/or sunshine hours on the incidence of specific diseases, using the odds ratio, relative risk, or percent changes of incidences, and using a large dataset with a span of 5–10 years. The rest of the studies ranked as low-quality since they only described seasonal patterns or annual trends of infectious diseases^98^^,^^112^^,^^138^ without measuring the specific effect of climate factors, and did not provide much practical information for developing early warning systems or other measures for infectious disease prevention. Climate-dengue association was found to be the most significant while the effects of climate factors on other infectious diseases were much slighter.^110^ The differences in methodology across studies also caused difficulties in comparing or identifying trends of climatic-infectious disease associations. For example, the meteorological data used varied across studies from daily data to weekly, monthly, quarterly, or even yearly data; and only a few studies analysed lag effects^101^^,^^106^^,^^107^^,^^111^^,^^116^^,^^119^^,^^130^^,^^132^ which led to heterogeneity of findings. Moreover, almost all studies did not account for non-environmental factors although infectious diseases themselves have diverse characteristics due to their modes of transmission and other environmental factors.

#### Health economic cost of climate change

There are two articles^76^^,^^139^ investigating the cost of the effects of climate change on health in Vietnam, and they only provided partial assessments. Nguyen 2021^139^ focused on the effects of temperature and precipitation changes on human migration and subsequent effects, including health expenditure, while Lohmann 2015^76^ focused on the effects of drought on health. The review found no specific study that estimates the costs associated with the impacts of climate change on human health in Vietnam. We estimated this cost using a “back-of-the-envelope” approach, which combines marginal effects parameters, projections of climate change impacts, GDP, and the population of Vietnam from various studies.^18^^,^^139^, ^140^, ^141^, ^142^, ^143^ A major effect of climate change is rising temperatures. We estimated that the healthcare costs of rising temperature due to climate change at present values is USD0.6 billion-USD2.0 billion. This figure may be a lower limit because it is expected that by 2035 Vietnam will face a population aging issue, which will result in higher health expenditure. In addition, the effects of climate change on health expenditure in Vietnam will involve increases ranging from USD1.0 billion to USD3.4 billion at present values. Apart from illness, climate change may also result in increased fatalities. The projected cost of climate change effects on premature deaths ranged from USD3 billion to USD20 billion. Heat-related illness also incurs costs of productivity loss due to work absenteeism. The estimated cost of work capacity loss due to climate changes varies from USD6 billion to USD23 billion by 2050 and will be the largest cost component.

### Awareness and engagement

Among ten papers that analysed awareness, communication, and participation in climate change and health, half examined the awareness and involvement of vulnerable populations, such as outdoor workers,^52^ people living in cramped shanty houses,^144^ children,^145^ farmers, and disadvantaged groups.^144^^,^^146^^,^^147^ Awareness levels of climate change and health impacts varied across studies.^144^^,^^148^, ^149^, ^150^, ^151^, ^152^ four indicated high awareness among community members and medical staff^144^^,^^148^, ^149^, ^150^ while two highlighted low levels of perception and interest in climate-related actions.^151^^,^^152^ A study among medical students exhibited a passive approach to learning about these topics, and the time allocated for these issues during the school year was limited.^149^ In addition, people were inadequately prepared for the adverse climate change impacts.^151^ Regarding factors influencing awareness, studies reported that males and people living in advantaged areas have a higher awareness of climate change and its impact on human health compared to females and those living in impoverished communities.^144^ In addition, high self-efficacy was positively correlated with the adoption of pro-environmental behaviour among school children.^145^ Studies also report a positive relationship between higher education and greater climate change awareness.^144^^,^^145^^,^^150^

There were high demands on communication and education programs to raise awareness and enhance adaptive skills in addressing the adverse effects of climate change, particularly climate change-related diseases.^52^^,^^151^ These studies recommend that communication content should also frame the simplest, most specific, and most effective adaptation and mitigation actions, such as disaster preparedness and health protection, and the economic and health benefits.^147^^,^^150^^,^^152^ Intervention programs on awareness, communication, and engagement with climate change and health should focus on vulnerable populations, such as those living in poor areas and with low levels of education.^52^^,^^151^ Additionally, establishing community groups as communication channels for climate change information, including prevention of seasonal epidemics and natural disasters, gained strong support.^144^ The author also suggested that future studies should consider employing environmental management experimental designs to provide precise guidance for effective communication practices.^145^

**Fig. 4:**
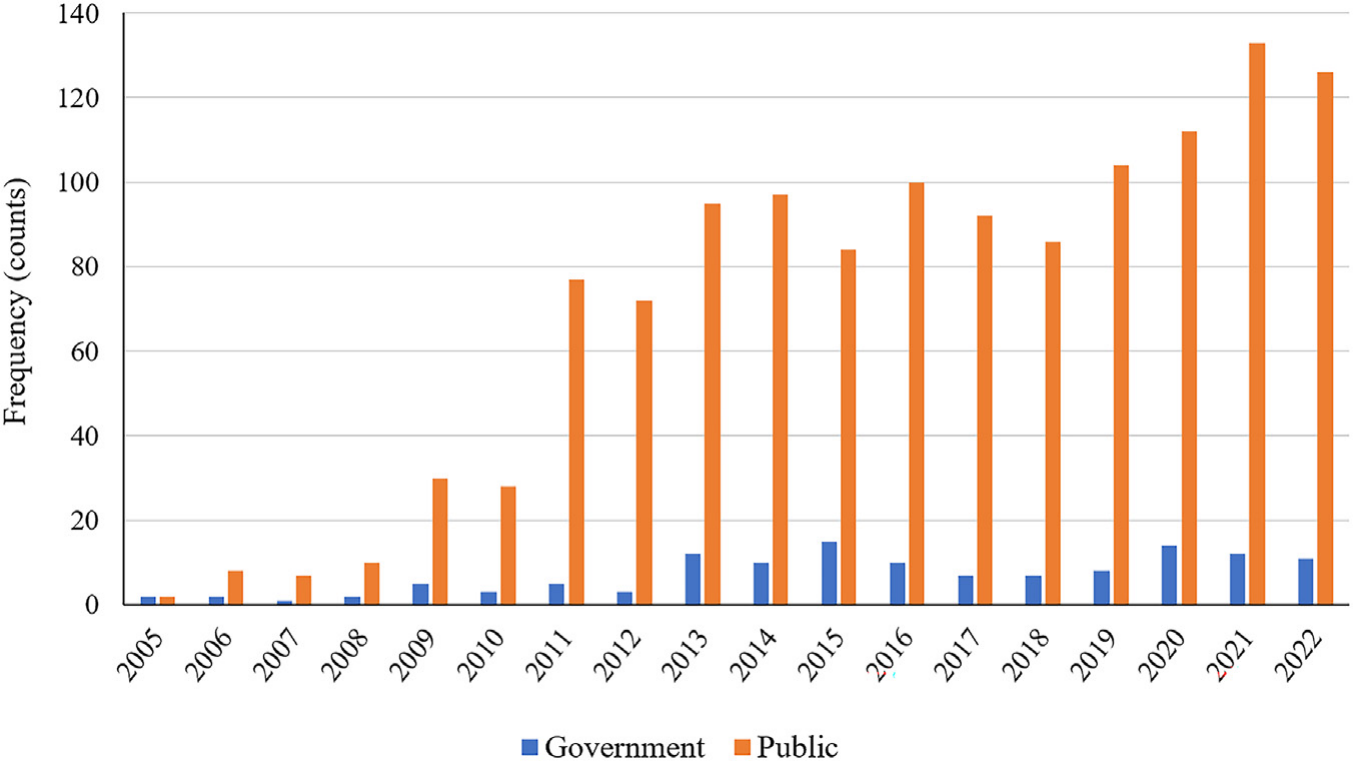
Frequency of media coverage on climate change and health covered in public media in Vietnam.

The additional analysis of the media coverage of the impact of climate change on human health from the Vietnamese government and public online media showed an increase in the number of both government and public articles over the years. The analysis found a total of 1392 media articles, 90.7% were from public media, with the remainder from government sources. Fig. 4 shows the media coverage on the topic from 2005 until 2022, broken down by year and type of media outlets. There was a significant increase in the number of media articles from 2008 (12) to 2011 (82). After 2013, the increase in media coverage remained relatively stable, with some fluctuation from year to year. Notably, while government article numbers remained low, public media articles have consistently increased.

### Climate Change Adaptation and Policies for Health

There were eight studies focusing on the adaptation of the community and the health sector to climate change. A comprehensive adaptation assessment in Vietnam in 2018 reported that the adaptive capacity of the health sector to climate change–related hazards was very low.^153^ An assessment in Ho Chi Minh City also highlighted that hospital safety in response to climate change was not high.^154^ Similar to this, Hoang et al. 2014^155^ reported that the primary care system in rural Vietnam was incapable of providing effective preventative and therapeutic healthcare in response to storm and flood-related health issues. Inadequate understanding of the complex impacts of climate change on the health of health staff, especially at provincial and primary health care levels were among the major obstacles in implementing action plans.^155^ Poor collaboration across institutions involved in climate change adaptation, and insufficient financial support and allocation were impediments to the health sector's ability to adapt.^156^^,^^157^

Concerning community adaptation, an assessment of the adaptation strategies for climate extreme events found that the majority of households did not employ any adaptation measures or implemented only reactive actions. Instead of proactively devising long-term adaptation plans, the community tended to respond to extreme events as they occurred.^158^ There were various barriers to adapting to and mitigating the impacts of extreme climate events, such as the low capacity of relevant staff, lack of local budgets, and outdated adaptation methods.^158^ The authors recommended that the adaptation process should focus on integrating vulnerability assessment findings appropriately with the assessment tools and gender analysis to develop and implement adaptation measures effectively and efficiently.^159^ Similarly, it is advised to maintain coastal ecosystem health which could aid daily living, and act as part of a climate adaptation strategy.^160^

Vietnam's climate change adaptation governance system consists of a comprehensive framework of policies for climate change adaptation, disaster risk management, and green growth with national, sub-national, and sectoral policies. The National Committee for Climate Change established in 2012 and chaired by the prime minister is the highest-level authoritative organisation in charge of formulating and implementing climate change policies. Since then, climate change-related legislation, strategies, and action plans have been launched under the Prime Minister and different ministries. Key policies include the *National climate change adaptation plan* for the 2021–2030 period with a vision by 2050 and the *National strategy for climate change until 2050*. A list of key policies and strategies for climate change adaptation in Vietnam from 1994 to 2022 was presented in Supplement #6.

In the health sector, the MOH holds responsibility for developing adaptation plans and policies. Accordingly, the MOH has issued a wide range of decisions, circulars, and guidelines related to climate change adaptation, including the *Action plan to respond to climate change in the health sector* for the period 2010–2015, and the *Action plan on disaster preparedness and the response of the health sector* for the period 2015–2020. Recently the MOH issued the *Climate change response action plan for the health sector* for the 2019–2030 period with a vision to 2050. The mechanisms and policies for the health sector's response to climate change, enhancing community awareness of, and capacity for, health protection and climate change adaptation, and strengthening the adaptability of health care systems, with a focus on the primary healthcare levels, were key climate change adaptation strategies for the health sector.^22^ The adaptation plan also emphasised the need to improve the database system for tracking, predicting, and providing early warning on how climate impacts on human health, as well as the need to prioritise developing a health care system that can meet the demand to prevent epidemics and emerging diseases brought on by climate change, with the focus on the MDR.^22^

The Vietnamese government has recognised the urgent need for adaptation strategies to address the health consequences of climate change impacts. The national government and the MOH have outlined health priorities and specific actions in their adaptation policies and plans which serve as a foundation for strengthening the resilience of the health system.^153^ However, Vietnam's climate change strategies are often based on national assessments and apply a top-down planning process.^153^ Therefore, sub-national plans are often just downscaled from national strategies, which limit the autonomy of local governments and are not based on local-specific context and priorities.^156^ In addition, the large number of legal documents related to climate change adaptation issued by the Prime Minister's office and other ministries lead to overlapping responsibilities and difficulties in implementation. The role and cooperation of the MOH with the National Committee for Climate Change and other related ministries and sectors are not emphasised.^157^ Existing regulations, mechanisms, and action plans in the health sector lack effective coordination in multi-disaster adaptation, integration between climate change adaptation and disaster risk reduction, and in addition fail to adequately address climate change-related health risks, especially heat-related health issues and post-disaster psychosocial problems.^156^^,^^157^

## Discussion

### Summary of the key findings

This is the most comprehensive systematic review on climate change and health in Vietnam. This paper has reviewed 127 journal articles, current climate change adaptation policies, and conducted four additional analyses on heatwave exposure, vulnerability, public and political engagement, and projection of climate change-related health costs in Vietnam by 2050.

The review findings indicate an increasing trend in the number of articles and an expanding range of study topics, especially since 2014, reflecting the rising concern about climate change and health in Vietnam. The studies consistently reported the effects of high temperature on mortality and hospitalisation risk, especially for climate-sensitive diseases such as infectious diseases, cardiovascular, respiratory, and kidney diseases. Also, rising temperatures and changing precipitation patterns were found to contribute significantly to the spread of outbreaks, especially of dengue fever and hand-foot-mouth disease. Other climate-related events such as floods, extreme rainfall, and drought were found to result in increased mortality, injuries, and hospitalisation risks. In the context of climate change, several potential biological mechanisms can contribute to adverse health impacts. These mechanisms can be multifaceted, involving physiological stress responses, immunological reactions, and cellular-level changes triggered by various climate-related exposures such as extreme heat, cold, or humidity.^1^^,^^7^ These mechanisms highlight the intricate ways in which climate change can influence human biology and health. Findings from reviewed studies provided evidence of the need for further adaptive intervention studies such as developing early warning systems for climate-sensitive disease prevention and providing communication and education programs on climate change and health.

The findings of the additional analyses demonstrate that heatwave exposure has consistently increased across all regions in Vietnam and the heat-health vulnerability of this country has been higher than the global average. These analyses suggest that future work should develop projection models to estimate the potential future changes in HEVI and EVPH indicators. Such an approach would offer a more comprehensive view, combining both historical trends and future projections. Furthermore, estimates of healthcare costs, premature deaths, and loss of work capacity associated with climate change by 2050 emphasise the significant costs and challenges faced by the health and economic sectors in the coming decades. The analysis of climate change and health articles in public media revealed a steady rise in the number of media articles over the years, reflecting an increasing interest and demand for information regarding the impact of climate change on human health. However, it is important to note that the number of government publications related to climate change and health remains relatively low compared to those from the citizen journalism and public domain. This highlights the necessity for greater involvement and active participation of governmental bodies in generating and disseminating research and policy-related publications.

The policy review indicated that the Vietnamese government and the MOH have recognised the urgent need for developing adaptation policies and action plans to strengthen the resilience of the health system and promote health outcomes for the population in the context of climate change. However, the adaptation capacity of the health system, especially at the primary level, has been relatively low.^153^^,^^155^ This threatened the achievement of the 2030 goal of universal healthcare, particularly in rural and mountainous areas characterised by lower health insurance coverage and high vulnerability to climate change.^161^ Without taking a climate change action plan, attaining the target of universal health care in Vietnam would be significantly more challenging. There were considerable obstacles in implementing climate change adaptation policies, especially in the health sector due to a lack of resources and inadequate multiple-sector coordination. One of the key challenges has been the top-down approach in policy development. This approach has resulted in a lack of consideration of local-specific context and priorities and has restricted the autonomy of local governments in developing local strategies. In addition, the MOH's role in national climate change adaptation strategies has been unrecognised, and overlapping responsibilities coupled with insufficient intersectoral collaboration have hindered the formulation of effective action plans. Finally, inadequate knowledge of climate change and health among health staff at both provincial and primary healthcare levels, coupled with limited budget allocation for health adaptation, have posed a major constraint in implementing prioritised adaptation options outlined in the plans.^162^

### Limitations and gaps of the previous studies

Prior research on climate change and health in Vietnam has several limitations. Inconsistencies in the quality and methodology across the reviewed studies, coupled with a limited number of long-term studies, obstructed a clear understanding of climate effects. Furthermore, the scant research on health-related economic costs and restrictions in the scope and settings of the studies made it difficult to understand the scale and comprehensive impacts of climate change. Concerning the quality of studies, our review showed that nearly two-thirds of the studies were ranked with low scores for quality or were classified as “cannot assess”. These low-quality studies often applied cross-sectional designs and conducted community surveys on a small-scale setting. Also, these studies often relied on self-reported health outcomes, implemented rapid assessments post-disasters, applied indirect health estimations, and used unclear methodology. These limitations introduced recall biases, hindered the generalisation of findings, restricted their practical relevance, and raised concerns about data accuracy and reliability. Additionally, differences in measuring exposure and health outcomes across studies made it challenging to compare their findings. Even the studies that attained higher scores of qualities are not exempt from limitations. Most studies relying on hospitalisation and meteorological data draw from limited sources, primarily large hospitals at the provincial or central level and central monitoring stations. Consequently, these studies fail to represent remote areas adequately. The restricted access to large datasets, limited data sharing across sectors, and technological challenges in storing electronic health data, particularly at the primary healthcare system level, presented significant obstacles to conducting robust studies in Vietnam.

This review also raises concerns over the limitations in study settings and research topics related to climate change and health in Vietnam. Most current studies have focused on single locations or big cities such as Ho Chi Minh, Hanoi, and Can Tho whereas there was a very limited number of studies in rural and mountainous areas, particularly in the Northern and Central Highlands regions, which have limited adaptive capacity to climate change. In addition, potential contributing factors such as socioeconomic and other health-related factors were often overlooked or insufficiently analysed. For example, the ageing population, environmental pollution, social deprivation, and decreasing public expenditure on healthcare and social welfare may aggravate the inequality of climate change-related health issues. In addition, some disasters such as floods, drought, salinity intrusion, and water quality deterioration are affected by both climate change and urbanisation phenomena such as population increase, construction of hydropower dams, and land use change. Therefore, it is a challenge to estimate the health burden attributable to climate change. Notably, the existing literature has exhibited the absence of future projections on climate-related health risks, particularly considering the different RCP scenarios and spatiotemporal variations across Vietnam. Adopting a more systematic approach in future studies to explore the variability of health impacts under different RCP scenarios, incorporating both quantitative modelling and longitudinal analysis, could bring more benefits. Moreover, numerous research topics on climate change and health remain unexplored. These include but are not limited to, intersecting climate risks, zoonotic diseases, long-term health outcomes and quality of life associated with climate change, as well as evaluations of the effectiveness of climate change adaptation measures.

### Recommendations

Based on evidence obtained from this current review we provide some recommendations for the research agenda and adaptation policy, focusing on improvement of research quality, future studies needed, and adaptation strategies. To enhance the quality of studies in this field, it is crucial to standardise measurements of climatic factors, health outcomes and methods for quantifying the effects, assessing vulnerability, and evaluating the effectiveness of adaptive capacity measures. These standardisations should be adopted by all scientists in the field. This process would maximise the effectiveness of validating and using the scientific evidence to inform policies and practices on climate change and health. In addition, promoting data sharing across sectors and groups of scientists would play an important role in improving research quality. It would give researchers the opportunity to share and use reliable and large datasets to improve the validity of their scientific evidence. This would also foster a multidisciplinary approach to address complex challenges in climate change adaptation across the sectors. A better understanding of the roles of potential confounding factors and effect modifiers would also help to improve the research quality in this field. Evaluations of internal and external validity of the evidence should also be considered seriously, and this process needs to be involved from the designing stage through to the completion of a study.

As for future research needs, more studies on multiple locations which are representative of the diversity of environmental, socioeconomic and population characteristics should be implemented. Multiple-location studies would not only improve the generalizability of the evidence and help us to better understand the interactive roles of other factors but also effectively inform the adaptive policy and practices in relevant geographical areas. Research in vulnerable communities which bear a disproportionate burden of the health impacts of climate change due to socioeconomic disparities and limited adaptive capacities should also prioritised. Additionally, exploring diverse climate-related hazards (such as heatwaves, extreme colds, droughts, wildfires, floods, storms, and salinisation) and assessing their interactions with concurrent crises, such as pandemics, is crucial for developing integrated response strategies.

Since climate change has already occurred and is on-going, it is timely to conduct studies on adaptation interventions to reduce the health impact of climate change-related factors while the mitigation measures are slowly progressed. Future studies should apply intervention approaches and implementation science to developing and evaluating climate-sensitive disease early warning systems, adaptation strategies and measures such as heat-health alert systems and action plans. The long-term effects of climate change on health outcomes, including mental health and non-communicable diseases, also need more studies. In addition, questions around the complex interactions between climate change and other factors such as socioeconomic factors, zoonoses, and health systems should be addressed. Establishment of a climate-health community of practices to gather multidisciplinary experts who share common research interests and best practices as well as creating new knowledge on research and practices would help promote the research in this field. Increasing funding opportunities for climate change and health studies from both governmental and private funding bodies would play an important role in encouraging scientists to conduct research on climate change and health.

The challenges in implementing climate change adaptation policies highlight the need for more inter-ministerial coordination and stronger efforts to integrate climate change considerations into policies and practices of the health and other sectors. Priorities identified in the health sector should be supported by other sectors to effectively address aspects of climate change and health. In addition, climate change adaptation strategies in the health sector need to take into account the burden of diseases, vulnerable population characteristics, and resources available in each region, province, and city. To ensure the success of climate change adaptation policies, especially the *Climate change response action plan of the health sector* in the 2019–2030 period, vision to 2050, it is crucial to monitor the implementation and assess the effectiveness of any plans. Also, it is advisable to take into account vulnerability and adaptation assessments for sectors that impact health, such as water, food security, and agriculture. These sectors serve as crucial entry points for the coordinated evaluation of health risks associated with climate change. Moreover, such assessments should strengthen scientific research about the health risks of climate change, vulnerable groups, and adaptation measures to enable policymakers to make necessary adjustments, including allocating adequate human and financial toward priority activities.

## Contributors

DP developed the concept and objectives for the study. All 23 authors met two times before starting the study to discuss about plan and task assigned for each person. The authors formed six working groups responsible for synthesising, analysing, and writing the initial manuscript for specific outcomes. Working Group 1 (Heat & Health studies) was led by DP, working Group 2 (climate-sensitive infectious diseases) was led by XHN, working Group 3 (extreme weather events and health impacts) was led by NQLT, working Group 4 (climate-related health economic lost) was led by SN, working Group 5 (climate change adaptation/policies) was led by TTTH, working group 6 (Awareness, communication and engagement) was led by LML and VB. DP planned and coordinated all activities of the Commission, the development and review of the report drafts, and the preparation for external peer review. DP, NQLT, CTP, and HTCHL reviewed and edited all sections of this report. In the article screening stage, the titles and abstracts were screened by KAD, HTCHL, VTN, THTD, HKTN, NQLT, and CTP, and then full-text reviews were performed by KAD, HTCHL, XHN, NQLT, TMLL, and CTP. In the analysing and writing stage, DP wrote the first and subsequent drafts of the Introduction and Methodology section, with input from NQLT, CTP, LHTCH. For Section 3.2.1 Impacts of temperatures on health, NDT wrote the first drafts, with input from DP, CTP, TTV, VQD, TNT, TNH. For Section 3.2.2 Impacts of extreme weather events, NQLT wrote the first and subsequent drafts, with input from HTCHL, PKT, and TATD. For Section 3.2.3 Climate-sensitive infectious diseases, XHN wrote the first and subsequent drafts, with input from NQLT, DP, and CTP. For Section 3.2.4 Health economic cost of climate change, SN wrote the first draft, with subsequent drafts written and edited by DP and CTP, with input from XBT, KN. For Section 3.3 Awareness and Engagement, LML and VB wrote the first draft, with subsequent drafts written and edited by TMLL, KAD, and CTP, with input from THTD, HKTN, VQD, KN. For Section 3.4 Climate Change Adaptation and Policies for Health, TTTH wrote the first and subsequent drafts, with input from CTP, NQLT, NHN, MCD, NTT. The entire manuscript was reviewed and revised by all 23 authors before submission. After receiving the reviewers’ comments, NQLT, HTCHL, CTP have accessed and verified the manuscript, DP were responsible for the decision to resubmit the manuscript.

## Editor note

The Lancet Group takes a neutral position with respect to territorial claims in published maps and institutional affiliations.

## Declaration of interests

All authors declare no competing interests.

## Appendix A Supplementary data

Supplement

Translated Abstract
