# Supplementary material for: Climate change and human health in Vietnam: a systematic review and additional analyses on current impacts, future risk, and adaptation
Source: Lancet Reg Health West Pac. 2023 Nov 15;40:100943. doi: 10.1016/j.lanwpc.2023.100943 (PMC10730327; doi:10.1016/j.lanwpc.2023.100943)

# **Supplemental #1. Translated abstract**

**Editors note:** *This translation in Vietnamese was submitted by the authors and we reproduce it as supplied. It has not been peer reviewed. Our editorial processes have only been applied to the original abstract in English, which should serve as reference for this manuscript.*

**Tóm tắt**

Nghiên cứu này nhằm mục đích đánh giá tác động của biến đổi khí hậu (BĐKH) đến sức khỏe và khả năng thích ứng ở Việt Nam thông qua xem xét tổng quan có hệ thống và tiến hành các phân tích bổ sung về nguy có phơi nhiễm với nhiệt độ cao, tính dễ bị tổn thương do nhiệt, nhận thức về BĐKH và sự tham gia trong thích ứng với BĐKH cũng như ước tính chi phí y tế liên quan đến BĐKH.

Trong số 127 nghiên cứu được xem xét, các phát hiện cho thấy sự lây lan rộng hơn của các bệnh truyền nhiễm, đồng thời tăng nguy cơ tử vong và nhập viện liên quan đến nắng nóng khắc nghiệt, hạn hán và lũ lụt. Tuy nhiên, có rất ít nghiên cứu đề cập đến chi phí y tế, nhận thức, sự tham gia, khả năng thích ứng và chính sách.

Các phân tích bổ sung cho thấy mức độ phơi nhiễm với sóng nhiệt ngày càng gia tăng trên khắp Việt Nam và mức độ dễ bị tổn thương trước nhiệt độ trên mức trung bình toàn cầu. Đến năm 2050, dự kiến biến đổi khí hậu sẽ gây thiệt hại lên tới 1-3 tỷ USD cho chi phí chăm sóc sức khỏe, 3-20 tỷ USD cho trường hợp tử vong sớm và 6-23 tỷ USD cho tình trạng mất việc làm.

Mặc dù các phương tiện truyền thông ngày càng tập trung vào khí hậu và sức khỏe, các bản tin đến từ công chúng nhiều hơn từ cơ quan chính phủ làm nổi bật sự cần thiết phải có sự tham gia nhiều hơn của chính phủ. Các chính sách thích ứng với BĐKH của Việt Nam phải đối mặt với những thách thức khi thực hiện, bao gồm cách tiếp cận từ trên xuống, thiếu hợp tác giữa các ban ngành, năng lực thích ứng với BĐKH thấp và nguồn lực hạn chế.

# **Supplemental #2. Search strategy**

## **For English papers**

| Database | Search term |
| --- | --- |
| Pubmed | ("Vietnam"[Mesh] OR Vietnam*****[tiab] OR "Mekong Delta"[tiab] OR "Southeast Asia*****"[tiab]) AND ("Climate Change"[Mesh] OR "Greenhouse Effect"[Mesh] OR climate[tiab] OR "climate change"[tiab] OR "global warming"[tiab] OR "climatic change*****"[tiab] OR "environmental change*****"[tiab] OR "greenhouse effect*****"[tiab] OR "carbon emission*****"[tiab] OR "climate disaster*****"[tiab] OR "climate variability"[tiab] OR "climatic variability"[tiab] OR "climate related"[tiab] OR weather[tiab] OR temperature[tiab] OR "temperature change"[tiab] OR "heat exposure"[tiab] OR "heat stress"[tiab] OR "heat strain"[tiab] OR "heat exhaustion"[tiab] OR "heat stroke"[tiab] OR "heat event*"[tiab] OR heatwave*[tiab] OR heat-wave*[tiab] OR humid*[tiab] OR precipitation[tiab] OR rain*[tiab] OR humid*[tiab] OR season*[tiab] OR storm*****[tiab] OR flood*****[tiab] OR flooding[tiab] OR drought*****[tiab] OR "forest fire*"[tiab] OR wildfire*[tiab] OR "wild fire*"[tiab] OR bushfire*[tiab] OR "bush fire*"[tiab] OR  "extreme weather"[tiab] OR "extreme heat*"[tiab] OR tornado*[tiab] OR blizzard[tiab] OR hail[tiab] OR hurricane*[tiab] OR cyclon*[tiab] OR monsoon*[tiab] OR meteorological[tiab] OR "sea level*"[tiab]) AND (Health[tiab] OR disease*[tiab] OR morbidity[tiab] OR disease[tiab] OR hospital*[tiab] OR illness*[tiab] OR "water-borne disease*"[tiab] OR "vector-borne disease*"[tiab] OR "climate disease*"[tiab] OR infect*[tiab] OR mortality[tiab] OR death*****[tiab] OR injur*[tiab] OR mental*[tiab] OR wellness[tiab] OR wellbeing[tiab])  Filters: from 2000/1/1 - Present |
| Embase | ('Viet Nam'/exp OR Vietnam*:ti,ab OR 'Mekong Delta':ti,ab OR 'Southeast Asia*':ti,ab) AND ('Climate Change'/exp OR 'Greenhouse Effect'/exp OR climate:ti,ab OR 'climate change':ti,ab OR 'global warming':ti,ab OR 'climatic change*':ti,ab OR 'environmental change*':ti,ab OR 'greenhouse effect*':ti,ab OR 'carbon emission*':ti,ab OR 'climate disaster*':ti,ab OR 'climate variability':ti,ab OR 'climatic variability':ti,ab OR 'climate related':ti,ab OR weather:ti,ab OR temperature:ti,ab OR 'temperature change':ti,ab OR 'heat exposure':ti,ab OR 'heat stress':ti,ab OR 'heat strain':ti,ab OR 'heat exhaustion':ti,ab OR 'heat stroke':ti,ab OR 'heat event*':ti,ab OR heatwave*:ti,ab OR heat-wave*:ti,ab OR humid*:ti,ab OR precipitation:ti,ab OR rain*:ti,ab OR humid*:ti,ab OR season*:ti,ab OR storm*:ti,ab OR flood*:ti,ab OR flooding:ti,ab OR drought*:ti,ab OR 'forest fire*':ti,ab OR wildfire*:ti,ab OR 'wild fire*':ti,ab OR bushfire*:ti,ab OR 'bush fire*':ti,ab OR 'extreme weather':ti,ab OR 'extreme heat*':ti,ab OR tornado*:ti,ab OR blizzard:ti,ab OR hail:ti,ab OR hurricane*:ti,ab OR cyclon*:ti,ab OR monsoon*:ti,ab OR meteorological:ti,ab OR 'sea level*':ti,ab) AND (Health:ti,ab OR disease*:ti,ab OR morbidity:ti,ab OR disease:ti,ab OR hospital*:ti,ab OR illness*:ti,ab OR 'water-borne disease*':ti,ab OR 'vector-borne disease*':ti,ab OR 'climate disease*':ti,ab OR infect*:ti,ab OR mortality:ti,ab OR death*:ti,ab OR injur*:ti,ab OR mental*:ti,ab OR wellness:ti,ab OR wellbeing:ti,ab)  **AND [embase]/lim** |
| Web of Science | ((TI=Vietnam* OR AB=Vietnam*) OR (TI="Mekong Delta" OR AB="Mekong Delta") OR (TI="Southeast Asia*" OR AB="Southeast Asia*"))  AND  ((TI=climate OR AB=climate) OR (TI="climate change" OR AB="climate change") OR (TI="global warming" OR AB="global warming") OR (TI="climatic change*" OR AB="climatic change*") OR (TI="environmental change*" OR AB="environmental change*") OR (TI="greenhouse effect*" OR AB="greenhouse effect*") OR (TI="carbon emission*" OR AB="carbon emission*") OR (TI="climate disaster*" OR AB="climate disaster*") OR (TI="climate variability" OR AB="climate variability") OR (TI="climatic variability" OR AB="climatic variability") OR (TI="climate related" OR AB="climate related") OR (TI=weather OR AB=weather) OR (TI=temperature OR AB=temperature) OR (TI="temperature change" OR AB="temperature change") OR (TI="heat exposure" OR AB="heat exposure") OR (TI="heat stress" OR AB="heat stress") OR (TI="heat strain" OR AB="heat strain") OR (TI="heat exhaustion" OR AB="heat exhaustion") OR (TI="heat stroke" OR AB="heat stroke") OR (TI="heat event*" OR AB="heat event*") OR (TI=heatwave* OR AB=heatwave*) OR (TI=heat-wave* OR AB=heat-wave*) OR (TI=humid* OR AB=humid*) OR (TI=precipitation OR AB=precipitation) OR (TI=rain* OR AB=rain*) OR (TI=humid* OR AB=humid*) OR (TI=season* OR AB=season*) OR (TI=storm* OR AB=storm*) OR (TI=flood* OR AB=flood*) OR (TI=flooding OR AB=flooding) OR (TI=drought* OR AB=drought*) OR (TI="forest fire*" OR AB="forest fire*") OR (TI=wildfire* OR AB=wildfire*) OR (TI="wild fire*" OR AB="wild fire*") OR (TI=bushfire* OR AB=bushfire*) OR (TI="bush fire*" OR AB="bush fire*") OR (TI="extreme weather" OR AB="extreme weather") OR (TI="extreme heat*" OR AB="extreme heat*") OR (TI=tornado* OR AB=tornado*) OR (TI=blizzard OR AB=blizzard) OR (TI=hail OR AB=hail) OR (TI=hurricane* OR AB=hurricane*) OR (TI=cyclon* OR AB=cyclon*) OR (TI=monsoon* OR AB=monsoon*) OR (TI=meteorological OR AB=meteorological) OR (TI="sea level*" OR AB="sea level*"))  AND  ((TI=Health OR AB=Health) OR (TI=disease* OR AB=disease*) OR (TI=morbidity OR AB=morbidity) OR (TI=disease OR AB=disease) OR (TI=hospital* OR AB=hospital*) OR (TI=illness* OR AB=illness*) OR (TI="water-borne disease*" OR AB="water-borne disease*") OR (TI="vector-borne disease*" OR AB="vector-borne disease*") OR (TI="climate disease*" OR AB="climate disease*") OR (TI=infect* OR AB=infect*) OR (TI=mortality OR AB=mortality) OR (TI=death* OR AB=death*) OR (TI=injur* OR AB=injur*) OR (TI=mental* OR AB=mental*) OR (TI=wellness OR AB=wellness) OR (TI=wellbeing OR AB=wellbeing))  Filter publish year and exclude conference titles |
| CINAHL | ((MH Vietnam+) OR (TI Vietnam* OR AB Vietnam*) OR (TI "Mekong Delta" OR AB "Mekong Delta") OR (TI "Southeast Asia*" OR AB "Southeast Asia*")) AND ((MH "Climate Change+") OR (MH "Greenhouse Effect+") OR (TI climate OR AB climate) OR (TI "climate change" OR AB "climate change") OR (TI "global warming" OR AB "global warming") OR (TI "climatic change*" OR AB "climatic change*") OR (TI "environmental change*" OR AB "environmental change*") OR (TI "greenhouse effect*" OR AB "greenhouse effect*") OR (TI "carbon emission*" OR AB "carbon emission*") OR (TI "climate disaster*" OR AB "climate disaster*") OR (TI "climate variability" OR AB "climate variability") OR (TI "climatic variability" OR AB "climatic variability") OR (TI "climate related" OR AB "climate related") OR (TI weather OR AB weather) OR (TI temperature OR AB temperature) OR (TI "temperature change" OR AB "temperature change") OR (TI "heat exposure" OR AB "heat exposure") OR (TI "heat stress" OR AB "heat stress") OR (TI "heat strain" OR AB "heat strain") OR (TI "heat exhaustion" OR AB "heat exhaustion") OR (TI "heat stroke" OR AB "heat stroke") OR (TI "heat event*" OR AB "heat event*") OR (TI heatwave* OR AB heatwave*) OR (TI heat-wave* OR AB heat-wave*) OR (TI humid* OR AB humid*) OR (TI precipitation OR AB precipitation) OR (TI rain* OR AB rain*) OR (TI humid* OR AB humid*) OR (TI season* OR AB season*) OR (TI storm* OR AB storm*) OR (TI flood* OR AB flood*) OR (TI flooding OR AB flooding) OR (TI drought* OR AB drought*) OR (TI "forest fire*" OR AB "forest fire*") OR (TI wildfire* OR AB wildfire*) OR (TI "wild fire*" OR AB "wild fire*") OR (TI bushfire* OR AB bushfire*) OR (TI "bush fire*" OR AB "bush fire*") OR (TI "extreme weather" OR AB "extreme weather") OR (TI "extreme heat*" OR AB "extreme heat*") OR (TI tornado* OR AB tornado*) OR (TI blizzard OR AB blizzard) OR (TI hail OR AB hail) OR (TI hurricane* OR AB hurricane*) OR (TI cyclon* OR AB cyclon*) OR (TI monsoon* OR AB monsoon*) OR (TI meteorological OR AB meteorological) OR (TI "sea level*" OR AB "sea level*")) AND ((TI Health OR AB Health) OR (TI disease* OR AB disease*) OR (TI morbidity OR AB morbidity) OR (TI disease OR AB disease) OR (TI hospital* OR AB hospital*) OR (TI illness* OR AB illness*) OR (TI "water-borne disease*" OR AB "water-borne disease*") OR (TI "vector-borne disease*" OR AB "vector-borne disease*") OR (TI "climate disease*" OR AB "climate disease*") OR (TI infect* OR AB infect*) OR (TI mortality OR AB mortality) OR (TI death* OR AB death*) OR (TI injur* OR AB injur*) OR (TI mental* OR AB mental*) OR (TI wellness OR AB wellness) OR (TI wellbeing OR AB wellbeing)) |

## **For Vietnamese papers in Google Scholar**

| Category | Search terms |
| --- | --- |
| 1. Biến đổi khí hậu (Climate change)  AND | “biến đổi khí hậu”, “nóng ấm toàn cầu”, “nóng lên toàn cầu”, “hiệu ứng nhà kính”, “phát thải các bon”, “thảm hoạ thiên nhiên”, “hiện tượng thời tiết khắc nghiệt”, “dgiao động thời tiết”, “sóng nhiệt”, “lạnh cực đoan”, “mưa cực đoan”, “hạn hán”, “rét đậm rét hại”, “lũ lụt”, “thiên tai”, “các yếu tố liên quan đến khí hậu” |
| 2. Sức khoẻ con người (Human health)  OR | “sức khoẻ”, “bệnh tật”, “sức khoẻ cộng đồng”, “gánh nặng bệnh tật”, “bệnh tật”, “tử vong”, “chấn thương”  “bệnh truyền qua véc tơ”, “bệnh do muỗi truyền”, “bệnh nhạy cảm với khí hậu”, “sức khoẻ tâm thần” |
| 3. Tính dễ bị tổn thương (Vulnerability)  OR | “dễ bị tổn thương”, “rủi ro”, “ các nhóm nhạy cảm”, “nguồn lực hạn chế”,  “thu nhập thấp”, “phơi nhiễm”, “tàn tật”, “khả năng hạn chế”, “khả năng tiếp cận”, “tính nhạy cảm”, “không được bảo vệ”, "tổn thương" |
| 4. Năng lực thích ứng (Adaptation)  OR | “giảm thiểu”, “biện pháp”, “thích ứng”, “đáp ứng”, “ứng phó”, “chiến lược”, “chính sách”, “kế hoạch thích ứng”, “kế hoạch ứng phó”, “năng lực thích ứng”, “năng lực ứng phó”, "thích nghi", "đối phó" |
| 5. Chính sách (policies) | “Văn bản quy phạm pháp luật”, “kế hoạch”, “chỉ thị”, “quyết định”, “nghị định”, “thông tư”, “luật”, “kế hoạch hành động” |

# **Supplemental #3. Included articles and data extraction.**

| **IDs** | **First author's name** | **Published Year** | **Title** | **Data period** | | **Study design** | **Focus topics** | **Regions** | **Climate variable(s)** | **Interested outcomes** | **Key findings** |
| --- | --- | --- | --- | --- | --- | --- | --- | --- | --- | --- | --- |
|  |  |  |  | **Start** | **End** |  |  |  |  |  |  |
| 1. **English papers** | | | | | | | | | | | |
| 1 | Althouse | 2018 | Seasonality of respiratory viruses causing hospitalizations for acute respiratory infections in children in Nha Trang, Vietnam | 2007 | 2013 | Longitudinal/Time-series | Climate-sensitive infectious diseases | South Central Coast | Temperature, rainfall, dew point, season | Acute Respiratory Infection (ARI) | Viral detection shows seasonality with peaks from May to September. Viral detection has a negative association with temperature and a positive association with dew point. RSV, Influenza A, and HPIV3 display significant seasonality; RSV peaks from July to November, and Influenza A peaks in May. Hospitalizations for ARI are negatively associated with temperature and positively associated with dew point. Influenza A and HRV have a negative association with temperature. HPIV3 and HRV have a positive association with dew point. Logistic regression reveals RSV is positively associated with the previous week's rain. The previous week's temperature is marginally associated with RSV. |
| 2 | Amstadter | 2009 | Posttyphoon prevalence of posttraumatic stress disorder, major depressive disorder, panic disorder, and generalized anxiety disorder in a Vietnamese sample | 2006 | 2006 |  | Extreme weather events | South Central Coast | Typhoons | Mental health | Post-typhoon prevalence rates for various disorders were as follows: PTSD 2.6%, MDD 5.9%, PD 9.3%, and GAD 2.2%. Among those who met the criteria for any disorder: 70% had only one disorder 15% had two disorders 14% had three disorders 1% met criteria for all four disorders |
| 3 | AnDTM | 2020 | Multilevel Analysis of 24-Hour Blood Pressure, Heart Rate, and Associated Factors among Police Officers in Hanoi, Vietnam. | N/A | N/A | Cross-sectional study | Temperature and health | Red River Delta | Temperature, humidity | Cause-specific morbidity | Temperature and humidity affected 74%-78% of Blood Pressure (BP) and Heart Rate (HR) variations among policemen. Increase in temperature or humidity led to a decrease in both Systolic and Diastolic BP. Time of day and seasonal transitions also influenced BP and HR variations. Individual factors like BMI and lifestyle contributed to 22%-26% of the variations. Traffic policemen were most affected by outdoor conditions. Recommendations include better uniforms and targeted training programs for police. |
| 4 | Bett | 2019 | Spatiotemporal analysis of historical records (2001-2012) on dengue fever in Vietnam and development of a statistical model for forecasting risk. | 2001 | 2012 | Longitudinal/Time-series | Climate-sensitive infectious diseases | Multiple regions | Temperature and precipitation | Dengue fever | Key predictors of dengue include minimum temperature, rainfall, altitude, and urban areas. Dengue incidence rises exponentially with temperature increases from 14 to 28.5˚C. Rainfall increases dengue incidence up to 550 mm, after which it declines. |
| 5 | Bich | 2011 | Impacts of flood on health: epidemiologic evidence from Hanoi, Vietnam. | 2008 | 2009 | Cross-sectional study | Extreme weather events | Red River Delta | Floods/rainfalls | Cause-specific morbidity | People in flood-prone areas face higher risks of psychological issues and communicable diseases like pink eye and dermatitis. Urgent need for effective flood prevention and mitigation strategies. |
| 6 | Bonell | 2020 | Does sunlight drive seasonality of TB in Vietnam? A retrospective environmental ecological study of tuberculosis seasonality in Vietnam from 2010 to 2015. | 2010 | 2015 | Longitudinal/Time-series | Climate-sensitive infectious diseases | Multiple regions | Temperature, rainfall, humidity, sunshine hours | Tuberculosis, Influenza-like-illness | 610,676 TB cases in Vietnam from 2010-2015, with notable seasonal and regional variations. Peaks in spring/summer, higher rates in the south, especially in Tay Ninh, An Giang, and Ho Chi Minh City. TB incidence linked to time, latitude, population density, youth percentage, and HIV prevalence. Climate factors like humidity, temperature, and sunlight also associated with TB incidence. |
| 7 | Bui | 2011 | Social and environmental determinants of malaria in space and time in Viet Nam. | 2007 | 2008 | Longitudinal/Time-series | Climate-sensitive infectious diseases | Multiple regions | Temperature and precipitation | Malaria | Incidence of reported malaria cases is highly spatially heterogeneous Poverty and forest cover are associated with malaria Incidence The effects of climate on malaria vary by socio-ecological zone. |
| 8 | Bulterys | 2013 | Environmental predictors and incubation period of AIDS-associated penicillium marneffei infection in Ho Chi Minh City, Vietnam. | 2004 | 2010 | Longitudinal/Time-series | Climate-sensitive infectious diseases | South East | Temperature, precipitation, humidity, visibility, wind speed | Hospitalization due to P. marneffei among HIV patients | P. marneffei admissions strongly linked to high humidity, not affected by precipitation, temperature, or wind. C. neoformans admissions not seasonal; P. marneffei more common during high humidity months. Estimated P. marneffei incubation period is 1 week. |
| 9 | Busby | 2018 | In harm's way: Climate security vulnerability in Asia | N/A | N/A | Multi-designs | Extreme weather events & Vulnerability assessment | Multiple regions | Multiple extreme weather events | vulnerability | Asia has a high risk of climate-related hazards and a high risk of climate-related death.  Floods, droughts, and storms are the most common disasters Vietnam had a governance score of 3 (1 is the worst and 7 is the best) Vietnam has a high score of hazard exposure in Asia and is among the top ten in the world for climate risk |
| 10 | Chen | 2020 | Flood impact on Mainland Southeast Asia between 1985 and 2018-The role of tropical cyclones | 1985 | 2018 | Longitudinal/Time-series | Extreme weather events | Multiple regions | Floods/rainfalls | Mortality | Vietnam had the highest rate of floods, followed by Thailand, Myanmar, Cambodia, and Lao PDR.  tropical cyclone floods (TCFloods) made up nearly 30% of all floods in Vietnam. In Vietnam, flood mortality rate was 24.3%; 30% of TCFloods accounted for 64% of flood deaths. |
| 11 | Chu | 2022 | Relationships between short-term ambient temperature exposure and kidney disease hospitalizations in the warm season in Vietnam: A case-crossover study. | 2003 | 2015 | Case-control study | Temperature and health | Multiple regions | Temperature | Kidney diseases | per 1◦C increase in daily mean temperature at lag 0–6 days: ORs=1.07 (glomerular diseases), 1.06 (renal tubulo-interstitial diseases), 1.12 (chronic kidney disease), and 1.09 (urolithiasis) |
| 12 | Cuong | 2011 | Quantifying the emergence of dengue in Hanoi, Vietnam: 1998-2009. | 1999 | 2008 | Time-series analysis | Climate-sensitive infectious diseases | Red River Delta | rainfall, wind velocity, temperature, and humidity | Dengue fever | Significant annual increase in dengue cases in Hanoi from 1999–2008, with an incidence rate ratio of 1.38. Median age of dengue cases is high at 23 years. Central, urban districts in Hanoi have the highest dengue incidence. annually outbreaks typically begin around July, peak in October, and then decrease towards the end of the year, corresponding with the wet and hot periods |
| 13 | Dang | 2019 | Effects of Extreme Temperatures on Mortality and Hospitalization in Ho Chi Minh City, Vietnam. | 2010 | 2013 | Longitudinal/Time-series | Temperature and health | South East | Temperature, dew point, and humidity | mortality (All-cause and cause-specifis) | The main effect of heatwaves significantly raise the risk of death from all causes and hospitalization Significant main effects were observed only for the elderly and people with respiratory issues. the main effect of heatwaves were higher than the added effect; in hospitalizations |
| 14 | Dang | 2016 | Characterizing the relationship between temperature and mortality in tropical and subtropical cities: a distributed lag non-linear model analysis in Hue, Viet Nam, 2009-2013. | 2009 | 2013 | Longitudinal/Time-series | Temperature and health | North Central Coast | Temperature | Mortality | Urban heat island effect contributes to 0.42% of heat-related issues. More green space can significantly reduce heat-related deaths. |
| 15 | Dang | 2018 | Green Space and Deaths Attributable to the Urban Heat Island Effect in Ho Chi Minh City. | 2010 | 2013 | Longitudinal/Time-series | Temperature and health | South East | Temperature |  | The urban heat island effect accounts for 0.42% of the problem, driven by varying temperatures between the two areas. For every additional square kilometer of green space per 1000 residents, 7.4 heat-related deaths can be avoided. |
| 16 | Dang | 2022 | Main and added effects of heatwaves on hospitalizations for mental and behavioral disorders in a tropical megacity of Vietnam. | 2017 | 2019 | Longitudinal/Time-series | Temperature and health | South East | Temperature | hospitalizations for mental and behavioral disorders | Heatwaves increase hospitalizations for mental and behavioral disorders (MBD) by 62%. Age groups are differently affected: 18-60 years by main heatwave effects, 61+ years by added effects. Substance use disorders and behavior-related conditions are especially vulnerable to heatwaves. High temperatures have both immediate and delayed effects on MBD hospitalizations. Gender and age influence the impact of heatwaves on MBDs. |
| 17 | Do | 2014 | Climatic-driven seasonality of emerging dengue fever in Hanoi, Vietnam. | 2002 | 2009 | Longitudinal/Time-series | Climate-sensitive infectious diseases | Red River Delta | Temperature, humidity, and precipitation | Dengue fever | Dengue fever (DF) incidence is linked to temperature, precipitation, and humidity A delayed impact of these factors on dengue incidence is observed, ranging from 8 to 18 weeks. The time delay between relative humidity and dengue incidence decreased from 14.30 to 5.27 weeks. |
| 18 | Do | 2022 | Impacts of Climate Change and Financial Support on Household Livelihoods: Evidence from the Northwest Sub-Region of Vietnam | 2021 | 2022 | Cross-sectional and qualitative study | Extreme weather events & Vulnerability assessment | North West | Multiple extreme weather events | vulnerability | Extreme weather events is increasingly affecting life expectancy, health, and household capital in Northwest Vietnam. Vulnerable groups like women and ethnic minorities have low adaptive capacity and are most affected. Microfinance significantly improves livelihood sustainability and reduces vulnerability among ethnic minority households. The study suggests specialized microfinance solutions for ethnic minority households to adapt to climate change impacts. |
| 19 | Few | 2013 | Seasonality, disease and behavior: using multiple methods to explore socio-environmental health risks in the Mekong Delta. | 2007 | 2008 | Multi-designs | Climate-sensitive infectious diseases & Vunerability assessment | Mekong River Delta | seasonality | diarrhoeal | No significant change in diarrheal disease risk due to high seasonality. Outcomes influenced by seasonal behaviors, household variation, and multiple disease pathways. Importance of social science in enhancing research depth noted. |
| 20 | Few | 2010 | Climatic hazards, health risk and response in Vietnam: Case studies on social dimensions of vulnerability | 2006 | 2006 | Multi-designs | Extreme weather events & Vulnerability assessment | Multiple regions | Multiple extreme weather events | self-reported of multi- diseases | Major health concerns in both communes are respiratory, gastrointestinal diseases, and dengue. Flooding significantly increases diarrhoeal disease risk, especially in children. Most people are aware that water contamination leads to infection risk. Environmental conditions also elevate the risk for skin diseases and conjunctivitis. In Cao Lanh and Long Xuyen, water-related diseases are a major concern; access to safe water varies from 40-60%. |
| 21 | Gan | 2021 | A scoping review of climate-related disasters in China, Indonesia and Vietnam: Disasters, health impacts, vulnerable populations and adaptation measures | 1992 | 2020 | Qualitative description (Scroping review) | Climate-sensitive infectious diseases & Vunerability assessment | Multiple regions | Multiple extreme weather events | climate-sensitive disease, malaria, injuries | Most studies on Vietnam's climate-related health impacts focus on "all-cause morbidity/mortality" and "infectious diseases"; few on heatstroke, injury, or PTSD. Common infectious diseases include dengue, diarrhea, and respiratory diseases. Poverty is the biggest risk factor for landslide vulnerability. Coastal residents are multi-hazard vulnerable; river basin residents are most vulnerable to floods. Vulnerable groups vary but often include the poor, rural communities, and those in fishing industries. |
| 22 | Gasparrini | 2017 | Projections of temperature-related excess mortality under climate change scenarios. | 2010 | 2019 | Longitudinal/Time-series | Temperature and health & Future risks | Multiple regions | Temperature | Heat-related, cold-related, and net excess mortality | Under all four scenarios, heat-related mortality in Vietnam is predicted to increase in the future. Cold-related mortality generally decreases across scenarios and time periods. The net effect shows an overall increase in mortality due to temperature changes, especially in the more severe scenarios. Scenario RCP8-5 projects the most dramatic increase, with a net mortality increase of up to +21.1 by 2090-2099. |
| 23 | Giang | 2014 | The effect of temperature on cardiovascular disease hospital admissions among elderly people in Thai Nguyen Province, Vietnam. | 2008 | 2012 | Longitudinal/Time-series | Temperature and health | North East | Temperature | cardiovascular diseases | The study identifies 26°C as the optimal temperature for minimum CVD admissions. Risk of CVD admissions increases both above and below this temperature, but effects are more pronounced and statistically significant for cold temperatures, peaking a week after exposure. No significant effects were found for hot temperatures or other weather variables on CVD admissions. |
| 24 | Gilfillan | 2017 | Coordination and health sector adaptation to climate change in the Vietnamese Mekong Delta | N/A | N/A | Multi-designs | Adaptation and Policy | Mekong River Delta | Climate change in general | three coordination dimensions: cross-scale, cross-sectoral, and cross-boundary | The study highlights three key coordination issues in adapting the Vietnamese Mekong Delta's health sector to climate change: cross-scale, cross-sectoral, and cross-boundary. Lack of collaboration between government bodies, top-down policy-making, and absence of formal mechanisms for interprovincial coordination inhibit effective adaptation. Despite challenges, the South West Steering Committee could facilitate better coordination. The findings are applicable not just to the Mekong Delta but to other sectors and regions in South East Asia. |
| 25 | Ho | 2018 | Retrospective analysis assessing the spatial and temporal distribution of paediatric acute respiratory tract infections in Ho Chi Minh City, Vietnam. | 2005 | 2010 | Longitudinal/Time-series | Climate-sensitive infectious diseases | South East | seasonality | Morbidity: Acute respiratory infections | Pediatric hospital admissions: 33% Acute Respiratory Infections (ARI),14% Upper Respiratory Infections (URI) and 19% Lower Respiratory Infections (LRI). Both URI and LRI showed significant increasing trends over time. Seasonal peaks were observed: URI in May–June and LRI in September–October.  These peaks were associated with weather factors like humidity, rainfall, and water level. |
| 26 | Ho | 2017 | A spatial and temporal analysis of paediatric central nervous system infections from 2005 to 2015 in Ho Chi Minh City, Vietnam. | 2005 | 2015 | Longitudinal/Time-series | Climate-sensitive infectious diseases | South East | seasonality, time trends | hospitalizations due to CNSI | Presumed bacterial infections (BI) were more common in the dry season and showed a decreasing trend. Both BI and non-BI were associated with changes in temperature, rainfall, and river water levels. |
| 27 | Hoang | 2022 | Assessing heat index changes in the context of climate change: A case study of Hanoi (Vietnam) | 1991 | 2020 | Longitudinal/Time-series | Temperature and health | Red River Delta | Heat index | Heat index | Heat index projected to rise by 0.0777°C/year under RCP 4.5 and 0.08°C/year under RCP 8.5. Weeks with dangerous heat levels will increase to 5.5 weeks every 5 years for RCP 4.5 and 6 weeks for RCP 8.5. Past incidence of heatstroke was rare, but will increase significantly in future scenarios, mainly in June, July, and August. |
| 28 | Huong | 2019 | Assessing household livelihood vulnerability to climate change: The case of Northwest Vietnam | 2015 | 2015 | Cross-sectional study | Extreme weather events & Vulnerability assessment | North East | Climate change in general | vulnerability index | "Pa Vay Su" community most vulnerable in multiple aspects including housing and health, while "Hien Luong" and "Moc Chau" had specific vulnerabilities. The vulnerability assessment indices can be applied in other areas and could form the basis for a national index for adaptation and mitigation needs. |
| 29 | Jalilov | 2018 | Sustainable Urban Water Management: Application for Integrated Assessment in Southeast Asia | N/A | 2017 | Cross-sectional study | Extreme weather events & Vulnerability assessment | Red River Delta | Climate change in general | water management | Future flood management should account for increased peak flows and flood inundation,  Should adopt flexible, adaptive measures due to uncertainties in climate and land use changes. |
| 30 | Kelly-Hope | 2008 | Temporal trends and climatic factors associated with bacterial enteric diseases in Vietnam, 1991-2001. | 1991 | 2001 | Longitudinal/Time-series | Temperature and health | Multiple regions | precipitation, temperature, and humidity | shigellosis, typhoid fever, and cholera | Bacterial enteric diseases in Vietnam show distinct temporal and seasonal trends, but their outbreaks are not strongly influenced by climate. Data quality issues like underreporting and misdiagnosis could also weaken the observed association with climate. |
| 31 | Kelly-Hope | 2007 | Geographical distribution and risk factors associated with enteric diseases in Vietnam. | 1991 | 2001 | Narative research | Climate-sensitive infectious diseases | Multiple regions | Temperature, rainfall, and vapor pressure values | shigellosis, typhoid fever, and cholera | Shigellosis is most prevalent in Central Highlands and correlates with rainfall and urban poverty. Typhoid is most common in the Mekong River Delta and is linked to vapor pressure and river/stream drinking water. Cholera is most prevalent along the Central Coastal regions and is associated with rainfall and public well drinking water. |
| 32 | Le | 2022 | The impacts of rainfall shocks on birth weight in Vietnam | 2004 | 2011 | Cross-sectional study | Extreme weather events | Multiple regions | Precipitation | birth weight | In-utero exposure to excessive or deficient rainfall during the second trimester leads to decreased birth weight. No impact is found from rainfall exposure in other trimesters. Vulnerability is higher among children from disadvantaged backgrounds, especially to deficient rainfall shocks. Infants born to poor, rural, and low-educated mothers are most at risk. |
| 33 | LeDang | 2014 | Farmers' perceived risks of climate change and influencing factors: a study in the Mekong Delta, Vietnam. | 2011 | 2012 |  | Awareness and Engagement | Mekong River Delta | Climate change in general | Climate change awareness | Farmers prioritize risks to production, physical health, and income over happiness and social relationships. Experience with climate change events increases perceived risks. Information sources can both increase or decrease perceived risks. Trust in public adaptation measures influences risk perception. Quality, timing, and channels of climate change information are crucial for managing risk perceptions. |
| 34 | Lee | 2019 | Predicted temperature-increase-induced global health burden and its regional variability. | 2010 | 2099 | Longitudinal/Time-series | Temperature and health & Future risks | Multiple regions | Temperature | Mortality | Findings for Vietnam: Temperature increase is 0.71°C. Heat Vulnerability: 11.82% increase per 1°C, Cold Vulnerability: -1.48% per 1°C, Total: 10.34%. Mortality Increase: 8.64% for heat, -1.02% for cold, Total: 7.62% per 100 ppm CO2. Vietnam, among other tropical countries, faces higher mortality risks with temperature increases. |
| 35 | Lee | 2017 | Seasonal and geographical distribution of bacillary dysentery (shigellosis) and associated climate risk factors in Kon Tam Province in Vietnam from 1999 to 2013. | 1999 | 2013 | Longitudinal/Time-series | Climate-sensitive infectious diseases | Multiple regions | Temperature, humidity, and precipitation | Bacillary dysentery (BD) | BD incidence slightly decreased over time, except a spike in 2012 in northern Vietnam. Higher rates in central regions, lower in northwest/east and Red River Delta. Peaks in mid and early/late rainy seasons. Higher risk in northwest during wet season (May-July). Rates increased May-October; in Kon Tum, temp, humidity, and precipitation positively linked to BD. |
| 36 | Lee | 2017 | Seasonal patterns of dengue fever and associated climate factors in 4 provinces in Vietnam from 1994 to 2013. | 1994 | 2013 | Longitudinal/Time-series | Climate-sensitive infectious diseases | Multiple regions | Temperature and precipitation | Dengue fever | Hanoi: 1°C temp rise led to 13% increase in Dengue Fever (DF); precipitation not significant. Khanh Hoa: 1°C temp rise and 100 mm precipitation increased DF by 17% and 11%, respectively. Ho Chi Minh City: No significant variables for DF. An Giang: 100 mm precipitation increased DF by 30% in preceding month and 22% in the same month. |
| 37 | Lohmann | 2015 | The Effect of Drought on Health Outcomes and Health Expenditures in Rural Vietnam | 2007 | 2013 | Cross-sectional study | Economic lost | Multiple regions | Drought | household welfare, health expenditures | Drought increases illness risk by 9%. Health expenditure rises by $144 or 10% of household consumption due to drought. Climate-related health costs go up 9-17% of household income. Direct link between drought and higher illness and healthcare costs. Severity of drought can lead to a 24% increase in household illness. Drought raises per-capita health expenses by about $115 or 11% of annual consumption. Impact varies by income; lower-income households see a 9% rise in health budget, while upper-income households see a 17% rise. |
| 38 | Lohrey | 2021 | Perceptions of heat-health impacts and the effects of knowledge and preventive actions by outdoor workers in Hanoi, Vietnam. | 2018 | 2018 | Cross-sectional study | Awareness and Engagement & Vulnerability assessment | Red River Delta | Climate change in general | heat-related impacts and vulnerabilities | Older females more likely to report symptoms and see doctor. Bedroom AC availability not a factor due to high costs. More knowledge of heat symptoms leads to more reporting and doctor visits. Checking weather updates correlates with more reported heat impacts and symptoms. Preventive actions correlate with reporting heat symptoms; drinking water doesn't help. Child carers and homeowners face income losses during heatwaves. |
| 39 | LuongLMT | 2019 | Effects of temperature on hospitalisation among pre-school children in Hanoi, Vietnam. | 2010 | 2014 | Longitudinal/Time-series | Temperature and health | Red River Delta | Temperature | Hospitalisation risk | 1°C drop in cold weather leads to 2.2% more respiratory hospital admissions for kids 3–5. 1°C increase in diurnal temp range in cold raises hospitalization 1.7-3.4% for kids < 5. Hot weather shows negative correlation with hospital admissions. Low temp and diurnal range in winter risk factors for hospitalization in kids < 5 in Hanoi. |
| 40 | Mabon | 2018 | Bringing social and cultural considerations into environmental management for vulnerable coastal communities: Responses to environmental change in Xuan Thuy National Park, Nam Dinh Province, Vietnam | N/A | N/A | Multi-designs | Adaptation and Policy | Red River Delta | Climate change in general | Adaptation | Xuan Thuy National Park is a vulnerable, environmentally-affected coastal area. Ecosystem health is vital for climate adaptation and livelihoods. Environmental changes impact well-being, social relations, health, and income. Technical competence is needed for coastal management. Socio-political responses are needed along with techno-scientific solutions. Environmental management is crucial for adaptation and cultural well-being, but structural issues must also be addressed. |
| 41 | Manabe | 2016 | Chronological, geographical, and seasonal trends of human cases of avian influenza A (H5N1) in Vietnam, 2003-2014: a spatial analysis. | 2003 | 2014 | Longitudinal/Time-series | Climate-sensitive infectious diseases | Multiple regions | seasonality | Human H5N1 case | A significant seasonal variation was observed, peaking in January and lowest in September, mainly from December to April. |
| 42 | Minh | 2020 | Adapting to Climate Extreme Events Based on Livelihood Strategies: Evidence from Rural Areas in Thua Thien Hue Province, Vietnam | 2019 | 2019 | Multi-designs | Adaptation and Policy | North Central Coast | Multiple extreme weather events | Adaptation | Dramatic fluctuation in climate events from 1999-2018 affects mainly agriculture. 44% reactive, 28% proactive adaptations; 27% no adaptation methods. Model explains 51.2% of factors affecting adaptation choices. Household behavior shows gap between perception of climate impacts and adaptation strategies. |
| 43 | MinhAn | 2014 | Epidemiology of dengue fever in Hanoi from 2002 to 2010 and its meteorological determinants. | 2002 | 2010 | Longitudinal/Time-series | Climate-sensitive infectious diseases | Red River Delta | Temperature, humidity, and precipitation | Dengue fever | 72.07% of DF cases in inner districts. Increasing annual and seasonal trend; low from Dec-Mar, peak in Sep-Oct. Significant relationship with rainfall at lag 1-3 months, temperature at lag 3 months. 92% correlation between predicted and observed dengue cases. |
| 44 | Mistry | 2022 | Comparison of weather station and climate reanalysis data for modelling temperature-related mortality | 1985 | 2019 | Longitudinal/Time-series | Temperature and health | Multiple regions | Temperature | Mortality | Vietnam: Fraction of all-cause excess mortality due to cold (2%) and heat (1.1%) Vietnam showed a significant divergence in heat-related RR from station observations and ERA5-Land, especially for extreme high temperatures. |
| 45 | Ngo | 2022 | Health trade-offs in pursuit of livelihood security: exploring the intersection of climate, migration and health from the perspective of Mekong Delta migrants in Ho Chi Minh City, Vietnam | 2019 | 2019 | Cross-sectional study | Extreme weather events & Vulnerability assessment | South East | Multiple extreme weather events | vulnerability | Weather variability damages homes, increases health risks and illnesses in Mekong Delta to Ho Chi Minh continuum. City faces storms, rising temps, more rainfall, flooding, and urban heat island effect. Migrants live in heat-stress, poorly-ventilated, flood-prone rental housing. |
| 46 | Ngo | 2022 | The persuasiveness of gain vs. loss framed messages on farmers' perceptions and decisions to climate change: A case study in coastal communities of Vietnam | 2021 | 2021 | Cross-sectional study | Awareness and Engagement | South Central Coast | Climate change in general | awareness | Gain-framed messages more effective than loss-framed in influencing risk perception and action on climate change, especially for farmers. Combining gain-framed with concrete messages yields best results for adaptation measures. |
| 47 | NgoHKT | 2021 | Impact of temperature on hospital admission for acute lower respiratory infection (ALRI) among pre-school children in Ho Chi Minh City, Vietnam. | 2013 | 2017 | Longitudinal/Time-series | Temperature and health | South East | Temperature | acute lower respiratory infection | 1°C max temp increase leads to 4.2% and 3.4% more ALRI hospital admissions in children 3-5 during dry and rainy seasons. 1°C DTR increase in rainy season reduces ALRI hospitalization risk by 2.0-2.5% for children <3. |
| 48 | Nguyen | 2020 | Impact of Climate Variability and Abundance of Mosquitoes on Dengue Transmission in Central Vietnam. | 1998 | 2009 | Longitudinal/Time-series | Climate-sensitive infectious diseases | Red River Delta | seasonality | Dengue fever | Dengue incidence peaks in autumn/winter; upward trend from 2004-2009. 42.9% of Hanoi districts show significant dengue clusters within 1,000m radius. Most cases from June-Nov, correlating with highest rainfall and temperatures. |
| 49 | Nguyen | 2020 | Temporal relationships between climate variables and hand-foot-mouth disease: a multi-province study in the Mekong Delta Region, Vietnam. | 2012 | 2014 | Longitudinal/Time-series | Climate-sensitive infectious diseases | Mekong River Delta | Temperature | HFMD | 1°C temp increase raises HFMD by 5.6% at lag 5 days 1% humidity increase boosts HFMD by 1.7% at lag 3 and 6 days. 1 unit more rainfall raises HFMD by 0.5% at lag 1 and 6 days. High HFMD rates in Thoi Lai, Binh Thuy, Ninh Kieu districts. |
| 50 | Nguyen | 2020 | Explaining Intentions by Vietnamese Schoolchildren to Adopt Pro-Environmental Behaviors in Response to Climate Change Using Theories of Persuasive Communication. | N/A | N/A | Cross-sectional study | Awareness and Engagement | Mekong River Delta | Climate change in general | awareness | Self-efficacy is key predictor of behavioral intention and climate risk prevention in schoolchildren;  attitude and subjective norm less impactful. |
| 51 | Nguyen | 2022 | Deep learning models for forecasting dengue fever based on climate data in Vietnam. | 1997 | 2016 | Longitudinal/Time-series | Climate-sensitive infectious diseases & Future risk | Multiple regions | Temperature, rainfall, humidity, evaporation, sunshine hours | Dengue fever | LSTM-ATT model best at forecasting DF incidence, outperforming others in 12/14 of 20 provinces for MAE or RMSE. Accurate up to 3 months ahead, but short-term forecasts slightly better. |
| 52 | Nguyen | 2017 | Temporal and spatial analysis of hand, foot, and mouth disease in relation to climate factors: A study in the Mekong Delta region, Vietnam | 2012 | 2014 | Longitudinal/Time-series | Climate-sensitive infectious diseases | Mekong River Delta | Temperature | HFMD | Mekong Delta vulnerable to climate change and HFMD. High temp, humidity, rainfall elevate HFMD risk. High-risk HFMD clusters in dense, high-traffic areas. Findings highlight climate's impact on health in MDR. |
| 53 | Nguyen | 2021 | Livelihood vulnerability to climate change in the mountains of Northern Vietnam: comparing the Hmong and the Dzao ethnic minority populations | 2017 | 2017 | Cross-sectional study | Extreme weather events & Vulnerability assessment | North East | Multiple extreme weather events | vulnerability | Hmong populations highly vulnerable to climate change impacts on housing, land, water, food, and health. Need for disaster risk and climate adaptation training for locals. |
| 54 | Nguyen | 2017 | Evaluating capacity for climate change adaptation in the health and water sectors in Vietnam: constraints and opportunities | 2010 | 2011 | Cross-sectional study | Adaptation and Policy | Red River Delta | Climate change in general | Adaptation | Top priorities: Training on vulnerability and increased funding for adaptation. Main challenges: Inadequate resources and weak cooperation. Governance issues like poor transparency exacerbate constraints on adaptive capacity in Vietnam. |
| 55 | Nguyen | 2021 | Do weather extremes induce people to move? Evidence from Vietnam | 2009 | 2014 | Longitudinal/Time-series | Economic lost | Multiple regions | Temperature and precipitation | Health economic lost | Low precipitation increases wage income by 7.9% but doesn't offset losses in other income sources. High precipitation reduces wage income, boosts agricultural income. Low and high temperatures reduce per capita income by 1.7% and 2.2%, respectively. High precipitation and temperatures increase out-patient healthcare visits and expenses. |
| 56 | Nguyen | 2021 | Predicting Future Urban Flood Risk Using Land Change and Hydraulic Modeling in a River Watershed in the Central Province of Vietnam | 1995 | 2040 | Incidence or prevalence study without comparison | Extreme weather events & Vulnerability assessment | South Central Coast | Floods/rainfalls | vulnerability | Flood risk was computed through hazard, exposure, and vulnerability and compared flood risk in 1995, 2019, and 2040. Flood risk increases with urbanization, population density, and more hospitals in flood plains. High and very high-risk areas decrease due to lower poverty rates. The study provides a framework for climate risk assessment and emphasizes the use of satellite data. |
| 57 | Nguyen | 2020 | A predictive model for dengue cáes in Southern Lamdong Province, Vietnam, 2007-2017 | 2007 | 2017 | Longitudinal/Time-series | Climate-sensitive infectious diseases | Central Highlands | Temperature, rainfall, humidity, wind velocity | Dengue fever | Significant correlation between monthly dengue cases, minimum temperature, and humidity. SARIMA model best for predicting dengue cases one month ahead. |
| 58 | Osti | 2011 | Lessons learned from statistical comparison of flood impact factors among southern and eastern Asian countries | 1980 | 2007 | Longitudinal/Time-series | Extreme weather events | Multiple regions | Floods/rainfalls | Mortality | Vietnam is one of the most impacted countries by floods in southern and eastern Asia;  Outdated disaster preparedness systems and lack of community capacity contribute to consistently high annual flood-related deaths. |
| 59 | Pham | 2009 | Correlates of environmental factors and human plague: an ecological study in Vietnam. | 1997 | 2002 | Longitudinal/Time-series | Climate-sensitive infectious diseases | Central Highlands | Temperature, rainfall, humidity, sunshine hours | Plague cases | 472 plague cases in Vietnam's Central Highlands from 1997-2002, with 5.1% fatality.  Cases peak in hot, dry months.  Risk factors include higher flea index, rodent density, and dry season. |
| 60 | Pham | 2011 | Ecological factors associated with dengue fever in a Central Highlands province, Vietnam. | 2004 | 2008 | Longitudinal/Time-series | Climate-sensitive infectious diseases | Central Highlands | Temperature, humidity, and precipitation | Dengue fever | Mosquito and climate factors drive dengue fever in Vietnam.  Global climate change may worsen the issue;  increased mosquito control during high temperature and rainfall seasons recommended. |
| 61 | Pham | 2022 | The child education and health ethnic inequality consequences of climate shocks in Vietnam | 2008 | 2017 | Cross-sectional study | Extreme weather events | Multiple regions | Floods/rainfalls | Children's health | Excess rainfall in the typhoon season negatively affects children's health and school enrollment, especially among ethnic minorities.  Last year's rainfall shocks impact current year's subjective health but not infectious disease incidence or grade progression.  Minority children are more affected than Kinh children by rainfall shocks. |
| 62 | PhamNTT | 2020 | Assessing and modelling vulnerability to dengue in the Mekong Delta of Vietnam by geospatial and time-series approaches. | 2001 | 2016 | Longitudinal/Time-series | Climate-sensitive infectious diseases & Vunerability assessment | Mekong River Delta | Temperature and precipitation | Dengue fever | Dengue vulnerability linked to climate, environment, and sociodemographics. Higher vulnerability in provinces along the Mekong River with urban and mixed horticulture land. Seasonal variation in vulnerability due to climate factors. Linear correlation between vulnerability value and dengue rates. Dengue rates correlated with rainfall. Time-series model predicts next year's vulnerability with average correlation of 0.69. |
| 63 | Phung | 2021 | Relationship between flood severity and risk of hospitalisation in the Mekong River Delta of Vietnam. | 2011 | 2014 | Longitudinal/Time-series | Extreme weather events | Mekong River Delta | river water level, flood severity | Hospitalisation risk | Extreme Annual Flood (2011) led to 7.2% rise in non-external cause hospitalizations, 16.4% in infectious diseases, and 25.5% in respiratory diseases. Incidence Rate Ratios showed 8.2% increase in respiratory disease hospitalizations. Hospitalizations decreased in dry season except for injuries. |
| 64 | Phung | 2015 | Climate change, water quality, and water-related diseases in the Mekong Delta Basin: a systematic review. | 2004 | 2015 | Qualitative description (review) | Climate-sensitive infectious diseases | Mekong River Delta | Multiple extreme weather events | water-related diseases | Diarrhea a major concern in Vietnam Mekong Delta. Typhoid fever linked to annual floods and temperature, not rainfall. Climate factors shape bacterial enteric disease patterns but not outbreaks. Typhoid most associated with vapor pressure and water quality in Mekong Delta. Malaria and dengue reemergence risk due to climate changes. Malaria cases peak October-January, often during El Nino disasters. Increased influenza tied to lower temperature, higher humidity, and peaks August-October. |
| 65 | Phung | 2017 | Heatwave and risk of hospitalization: A multi-province study in Vietnam. | 2002 | 2015 | Longitudinal/Time-series | Temperature and health | Multiple regions | Temperature (humidity, rainfall, and day of the week) | Hospitalisation risk | Country pooled effects: Heatwaves significantly affect all causes and infectious diseases on the same day. Northern Vietnam has higher heatwave-related hospitalization rates than the south. Females more sensitive to heatwaves in some southwestern provinces. Age and socio-economic factors show non-significant impact on heatwave sensitivity. |
| 66 | Phung | 2015 | Association between climate factors and diarrhoea in a Mekong Delta area. | 2004 | 2011 | Longitudinal/Time-series | Climate-sensitive infectious diseases | Mekong River Delta | Temperature, humidity, and precipitation | diarrhoeal | 1 unit increase in RWL (cm) leads to 0.07% increase in diarrhoeal rate. 1°C temperature rise in past 2-4 weeks increases diarrhoeal rates by 1.5% and 1.1% respectively. 1% rise in humidity decreases diarrhoeal rate by 0.4%. More diarrhoea clusters found in peri-urban areas than in rural areas. |
| 67 | Phung | 2015 | Temporal and spatial patterns of diarrhoea in the Mekong Delta area, Vietnam. | 2004 | 2011 | Longitudinal/Time-series | Climate-sensitive infectious diseases | Mekong River Delta | temperature, rainfall, humidity, river-water-level |  | Diarrhoea peaks between August-October annually. High temperature, humidity, and rainfall positively associated with increased diarrhoea. Climate-diarrhoea link stronger in rural areas than urban. |
| 68 | Phung | 2018 | Spatial variation of heat-related morbidity: A hierarchical Bayesian analysis in multiple districts of the Mekong Delta Region. | 2010 | 2013 | Longitudinal/Time-series | Temperature and health | Mekong River Delta | Temperature, humidity, and precipitation | Hospitalisation risk | 5°C rise in temperature leads to 6.1% increase in hospital admissions. District-level risks vary widely, from 55.2% decrease to 24.4% increase per 5°C temperature rise. Population density, female population, and preschool students increase temperature-hospitalization risk. Rural population decreases temperature-hospitalization risk. |
| 69 | Phung | 2016 | The spatial distribution of vulnerability to the health impacts of flooding in the Mekong Delta, Vietnam. | 2015 | 2015 | Case-cross over | Extreme weather events | Mekong River Delta | Floods/rainfalls | vulnerability | 29 indicators developed for vulnerability assessment. Vulnerability to flooding varies by province in Vietnam's Mekong Delta. Dong Thap and An Giang have highest vulnerability indexes. Higher vulnerability in upstream provinces near rivers compared to coastal areas. Principal component analysis aligns well with analytic hierarchy process method. |
| 70 | Phung | 2014 | Association between annual river flood pulse and paediatric hospital admissions in the Mekong Delta area. | 2008 | 2011 | Longitudinal/Time-series | Extreme weather events | Mekong River Delta | river water level | Hospitalisation risk | Cumulative risk ratios show increased risk following extreme daily river water levels for various age groups and causes. Peak risk for daily pediatric hospital admissions is in September-October, correlating with highest river water levels. Risk association between pediatric hospital admissions and river water levels modified by temperature. |
| 71 | Phung | 2015 | Temperature as a risk factor for hospitalisations among young children in the Mekong Delta area, Vietnam. | 2008 | 2012 | Longitudinal/Time-series | Temperature and health | Mekong River Delta | Temperature | Hospitalisation risk | Increased mean temperature linked to higher risk of pediatric hospital admissions (PHAs) for 0–2-year-olds on current and previous day, and for 3–5-year-olds with a 1-2 day lag. 1°C temperature rise on current and previous day increases PHA risk by 1.7% for 0–2-year-olds and 1.6% for 3–5-year-olds. 1°C temperature rise on current and previous day increases overall PHA risk by 1.3%. |
| 72 | Phung | 2017 | Heavy rainfall and risk of infectious intestinal diseases in the most populous city in Vietnam. | 2004 | 2013 | Longitudinal/Time-series | Climate-sensitive infectious diseases | South East | Floods/rainfalls | Hospitalisation risk | Heavy rainfall events (HREs) linked to increased hospital admissions for intestinal infectious diseases (IID), with a 13.2% rise observed 4–6 days after an HRE. Effects of HREs on IID vary among individual events. Statistically significant IID increases only seen for lags of 4-6 days, ranging from 12.9% to 13.5%. Average daily decrease of 0.11% in IID observed post-HRE |
| 73 | Phung | 2016 | High temperature and risk of hospitalizations, and effect modifying potential of socio-economic conditions: A multi-province study in the tropical Mekong Delta Region. | 2002 | 2014 | Longitudinal/Time-series | Temperature and health | Mekong River Delta | Temperature | Hospitalisation risk | 1°C rise in temperature increases hospital admissions by 1.3% for all causes, 2.2% for infectious diseases, and 1.1% for respiratory diseases; inconsistent results for cardiovascular diseases. Higher population density, poverty, and illiteracy increase hospitalization risk due to high temperature. Higher income and better sanitation reduce hospitalization risk due to high temperature. |
| 74 | Phung | 2018 | Spatiotemporal variation of hand-foot-mouth disease in relation to socioecological factors: A multiple-province analysis in Vietnam. | 2011 | 2014 | Longitudinal/Time-series | Climate-sensitive infectious diseases | Multiple regions | Temperature | Hospitalisation risk | 5°C rise in average temperature linked to 6.1% increase in hospital admissions. District-level admission changes vary widely, from a 55.2% decrease to a 24.4% increase per 5°C temperature rise. Temperature-hospitalization risk rises with higher population density, female population, and pre-school students. Higher rural population percentage reduces temperature-hospitalization risk. |
| 75 | Phung | 2016 | The effects of high temperature on cardiovascular admissions in the most populous tropical city in Vietnam. | 2004 | 2013 | Longitudinal/Time-series | Temperature and health | Multiple regions | Temperature | cardiovascular diseases | J-shape relationship between temperature and CHA, with a threshold at 29.6°C. Delayed effects of temperature on CHA last 0 to 5 days. Risk of CHA rises 12.9% during heatwave events (temperatures in the 99th percentile for 2 days). |
| 76 | Phung | 2018 | The effects of socioecological factors on variation of communicable diseases: A multiple-disease study at the national scale of Vietnam. | 2011 | 2015 | Longitudinal/Time-series | Climate-sensitive infectious diseases | Multiple regions | multi climate factors | Hospitalisation risk | Oral-transmission diseases spread nationwide; airborne diseases cluster in Northwest; vector-borne diseases cluster in South. All listed diseases linked to at least 2 of 3 climatic factors (temperature, humidity, rainfall). Dengue most sensitive to climate. Socio-economic influences vary; population density is the biggest contributor to disease risk. |
| 77 | Phung | 2021 | Hydropower dams, river drought and health effects: A detection and attribution study in the lower Mekong Delta Region | 1978 | 2014 | Longitudinal/Time-series | Extreme weather events | Mekong River Delta | Drought | Hospitalisation risk | River drought increased risk of all-cause, respiratory, and renal hospitalizations by 2%, 2%, and 7% respectively. 1134 all-cause hospitalizations from 1995–2014 due to drought, costing $360,385 at two provincial hospitals. |
| 78 | PhuongLTD | 2016 | Climate Variability and Dengue Hemorrhagic Fever in Ba Tri District, Ben Tre Province, Vietnam during 2004-2014. | 2004 | 2014 | Longitudinal/Time-series | Climate-sensitive infectious diseases | Mekong River Delta | temperature, (rainfall, humidity) | Dengue fever | 5728 reported DF/DHF cases and five deaths from 2004–2014. Cases peak from May to October, highest in June and July. Strong correlations with rainfall, humidity, mosquito density, and Breteau index. Moderate correlation with average temperature and Aedes mosquito density. |
| 79 | Pollack | 2016 | Mental health, life functioning and risk factors among people exposed to frequent natural disasters and chronic poverty in Vietnam. | 2013 | 2013 | Cross-sectional study | Extreme weather events | South Central Coast | Multiple extreme weather events | mental health | 22.7% met criteria for mental health issues. 22.1% reported functional impairment. 99% had lifetime exposure to major storms; 77% traumatic. 30% reported financial stress. Traumatic storms, not frequency, linked to mental health. Financial stress strongest mental health predictor. Traumatic storms predict financial stress (r2=0.03). |
| 80 | Rubin | 2014 | Social vulnerability to climate-induced natural disasters: Cross-provincial evidence from Vietnam | 2000 | 2009 | Longitudinal/Time-series | Extreme weather events | Multiple regions | Multiple extreme weather events | vulnerability | Socioeconomic factors explain Vietnam's regional disaster impact. Inequality, poverty, and infant mortality increase fatalities. Adaptation should address socioeconomic issues and geophysical risks. |
| 81 | Shi | 2016 | Mapping and ranking global mortality, affected population and GDP loss risks for multiple climatic hazards | 1980 | 2009 | Cross-sectional study | Extreme weather events | Multiple regions | Multiple extreme weather events | mortality, affected population and GDP loss | Vietnam is a hotspot for climate hazard High-risk countries have large populations or GDP. Reducing exposure and improving governance is key. Should focus on coping capacity in hotspots like the Philippines, Bangladesh, and Vietnam. |
| 82 | Talukder | 2022 | The effect of high temperatures on risk of hospitalization in northern Vietnam. | 2005 | 2015 | Longitudinal/Time-series | Temperature and health | Multiple regions | Temperature | Hospitalisation risk | 1°C increase above 24°C raises all-cause hospital admissions by 1.1%. Infectious disease admissions increase by 2.4%. Cardiovascular disease admissions increase by 0.5%. Respiratory disease admissions increase by 1.3%. |
| 83 | TalukderMRR | 2018 | Association between salinity and hospital admission for hypertension: an ecological case-control study in the Mekong Delta Region in Vietnam. | 2013 | 2013 | Case-control study | Extreme weather events | Mekong River Delta | salinity | hypertension (hospitalization) | 3.9% of cases were hypertensive. 9% increased risk of hypertension admission in exposed areas vs non-exposed. |
| 84 | Thai | 2015 | Seasonality of absolute humidity explains seasonality of influenza-like illness in Vietnam. | 1993 | 2010 | Longitudinal/Time-series | Climate-sensitive infectious diseases | Multiple regions | humidity | influenza-like illness | Influenza-like illness seasonality in Vietnam correlates with absolute humidity fluctuations. Biannual signals are considered less seasonal than annual ones. Higher elevation locations have stronger seasonality. |
| 85 | Thai | 2014 | Child Schooling, Child Health, and Rainfall Shocks: Evidence from Rural Vietnam | 1950 | 1998 | Cross-sectional study | Extreme weather events | Multiple regions | Floods/rainfalls | children's health | Positive rainfall shock speeds up school entry and progress; negative shock delays them. Rainfall shock affects schooling gap nonlinearly. Rainfall in utero year doesn't impact height-for-age. Positive rainfall in second year post-birth boosts height-for-age. Negative rainfall in third year harms schooling and height. |
| 86 | Thai | 2010 | Dengue dynamics in binh thuan province, southern Vietnam: Periodicity, synchronicity and climate variability | 1994 | 2019 | Longitudinal/Time-series | Climate-sensitive infectious diseases | South East | ENSO | Dengue fever | Strong link between ENSO, climate variables, and dengue in 2-3 year cycle. |
| 87 | ThiTuyet-Hanh | 2018 | Climate Variability and Dengue Hemorrhagic Fever in Hanoi, Viet Nam, During 2008 to 2015. | 2008 | 2015 | Longitudinal/Time-series | Climate-sensitive infectious diseases | Red River Delta | Temperature (humidity, rainfall) | Dengue fever | Monthly DF/DHF correlated with evaporation, negative with humidity, and positive with sunshine. No direct correlation with average rainfall and temperature. 3-month lag model: 1°C temp increase leads to 23.48% more DF/DHF cases; sunshine, humidity, rainfall, and evaporation also linked to DF/DHF increases 3 months later. |
| 88 | Thompson | 2015 | The impact of environmental and climatic variation on the spatiotemporal trends of hospitalized pediatric diarrhea in Ho Chi Minh City, Vietnam. | 2005 | 2010 | Longitudinal/Time-series | Climate-sensitive infectious diseases | South East | temperature, rainfall, humidity, river-water-level | diarrhoeal | Significant spatial differences in diarrhea risk across HCMC districts. Low elevation and varying responses to flooding, temperature, and humidity contribute to risk. Results useful for predictive algorithms to improve disease prevention. |
| 89 | ThuDang | 2019 | Short - term effects of temperature on hospital admissions for acute myocardial infarction: A comparison between two neighboring climate zones in Vietnam. | 2008 | 2015 | Longitudinal/Time-series | Temperature and health | Multiple regions | Temperature | acute myocardial infarction | North-Central Coast: 11% AMI HA risk increase at moderately low temps, 25% increase at extremely low temps. South-Central Coast: 18% AMI HA risk increase at moderately high temps, 36% increase at extremely high temps. |
| 90 | Toando | 2014 | Perceptions of climate change and its impact on human health: an integrated quantitative and qualitative approach. | 2012 | 2013 | Cross-sectional study | Awareness and Engagement | Red River Delta | Climate change in general | awareness | 79.3% in non-slum and 70.1% in slum areas aware of climate change's health impact. Different perceptions between non-slum and slum residents. Men, non-slum residents, and higher education linked to better climate change perception. No link between perception and socioeconomic status. Increased illness compared to past years. |
| 91 | Toando | 2013 | Hot spot detection and spatio-temporal dispersion of dengue fever in Hanoi, Vietnam. | 1998 | 2009 | Longitudinal/Time-series | Adaptation and Policy | Red River Delta | seasonality | Dengue fever | Geographic expansion of DF transmission in Hanoi (OR 1.17). 42.9% of Hanoi districts show significant DF clusters within a 1,000m radius for 29 days. Most DF cases from June-November, coinciding with highest rainfall and temperatures. |
| 92 | Tran | 2020 | Spatial patterns of health vulnerability to heatwaves in Vietnam. | 2005 | 2017 | Longitudinal/Time-series | Temperature and health & Vulnerability assessment | Multiple regions | Temperature | vulnerability | Heatwave vulnerability 'hotspots' identified in Southeast, Central Highlands, and South Central Coast of Vietnam. Hotspots not necessarily most vulnerable to climate change; adaptation capacity varies. Vulnerability index helps policymakers allocate resources and tailor regional strategies. |
| 93 | Tran | 2022 | The Association Between Ambient Temperatures and Hospital Admissions Due to Respiratory Diseases in the Capital City of Vietnam. | 2017 | 2018 | Longitudinal/Time-series | Temperature and health | Red River Delta | Temperature | espiratory diseases (hospitalization) | Lowest risk temperatures: 22-25°C. Cold temps riskier than hot across all demographics. Highest risk at 13°C for cold and 33°C for hot. Respiratory diseases most affected immediately by heat and 1-day lag by cold. Higher risk for women and those over 5 years old. |
| 94 | Tran | 2021 | Gaps in awareness of climate variability and its impacts on society among health professionals and community workers in Vietnam: Implications for COVID-19 and other epidemic response systems | 2020 | 2020 | survey | Awareness and Engagement | Multiple regions | Climate change in general | awareness | Participants have moderately high agreement in the impacts on health, society and economy and the impact on individuals and families The mean score of “Impacts on health, society and economy” and “Impact on individuals and families” are 7.9 and 7.6, out of 10) |
| 95 | Trang | 2016 | Heatwaves and Hospital Admissions for Mental Disorders in Northern Vietnam. | 2008 | 2012 | Longitudinal/Time-series | Temperature and health | Red River Delta | Temperature | mental health | Mental disorders risk increases with higher temps and longer heatwaves. Rural populations and men more at risk during 3-day heatwaves. Over-60s and rural residents most affected in 7-day heatwaves. Mental retardation and organic mental disorders show highest risk during specific heatwave durations. |
| 96 | Trang | 2016 | Seasonality of hospital admissions for mental disorders in Hanoi, Vietnam. | 2008 | 2012 | Longitudinal/Time-series | Temperature and health | Red River Delta | seasonality | mental health | 24% rise in mental disorder hospital visits in summer, peaking in June. Males and elderly (over 60) more sensitive to seasonal risk. 26% increase in admissions among men, 23% among the elderly in summer. |
| 97 | TranNQL | 2022 | The Prevalence of Heat-related Illnesses and Associated Factors among Rice Farmers in Vietnam. | 2021 | 2021 | Cross-sectional study | Temperature and health | North Central Coast | Temperature | Heat-Related Illness | 83.4% of farmers experienced at least one Heat-Related Illness (HRI) symptom; 55.1% experienced two or more last harvest season. Working over 4 hours in heat, heavy tasks, hypertension, and chronic conditions increase HRI risk. Older farmers and increased water intake associated with lower HRI risk. |
| 98 | Trinh | 2021 | Rainfall shocks and child health: the role of parental mental health | 1970 | 2014 | Longitudinal/Time-series | Extreme weather events & Vulnerability assessment | Multiple regions |  | parents' mental health, children weight | Rainfall shocks negatively affect parents' mental health, leading to increased likelihood of children being underweight. Households receiving support are less vulnerable to rainfall shocks' health impact. |
| 99 | Truong | 2019 | A spatial-temporal statistical analysis of health seasonality: explaining HFMD infections within a children population along the Vietnamese south central coast. | 2012 | 2016 | Longitudinal/Time-series | Climate-sensitive infectious diseases | South Central Coast | Temperature | HFMD | Two annual peaks in hand-foot-mouth disease in children: a large one in April and a smaller one during preschooling period. Disease peaks do not align with temperature peaks from January to May. Negative correlation between preschooling period and disease incidence from August to December. |
| 100 | Tu | 2011 | Adaptation to flood risks in Ho Chi Minh City, Vietnam | 2008 | 2008 | Qualitative description | Extreme weather events | South East | Floods/rainfalls | vulnerability | Rapid urbanization increases vulnerabilities to flood risks, affecting poor residents more. Binh Thanh District more flood-prone than District 2, despite better infrastructure. Poor people more vulnerable to floodwater pollution due to untreated water sources. Poor people's low awareness and capacity indirectly increase vulnerabilities for richer residents. |
| 101 | TuyetHanh | 2020 | Vietnam Climate Change and Health Vulnerability and Adaptation Assessment, 2018. | 2013 | 2018 | Multi-designs | Adaptation and Policy | Multiple regions | Climate change in general | vulnerability | Climate-sensitive diseases common and increasing. High exposure and sensitivity to climate hazards in health sector. Very low adaptive capacity, with overall high to very high risk from 2013-2017. |
| 102 | VanMinh | 2014 | Primary healthcare system capacities for responding to storm and flood-related health problems: a case study from a rural district in central Vietnam. | 2012 | 2012 | Cross-sectional study | Adaptation and Policy | North Central Coast | Multiple extreme weather events | Adaptation | Emergency services were not always available during disasters. Plans focus on response, not prevention, with unclear roles and coordination. Limited, slow funding for healthcare in disasters. Medical teams lack specialized training and experts. Insufficient data and early-warning systems. Inconsistent availability of emergency treatment protocols. |
| 103 | VanPham | 2019 | Meteorological factors associated with hand, foot and mouth disease in a Central Highlands province in Viet Nam: an ecological study. | 2012 | 2013 | Longitudinal/Time-series | Extreme weather events | Central Highlands | Temperature, humidity, and precipitation | HFMD | HFMD cases rise in the rainy season  HFMD is linked to higher temperatures (RR=1.06 per 1 °C increase), more rainfall (RR=1.19 per 200 mm increase), and longer sunshine (RR=1.14 per 60 hours increase) but decrease with higher wind speed (RR=0.77 per 1 m/s increase) |
| 104 | Vo | 2022 | Climate change and rural vulnerability in Vietnam: An analysis of livelihood vulnerability index | 2018 | 2018 | Cross-sectional study | Extreme weather events & Vulnerability assessment | Multiple regions | Multiple extreme weather events | vulnerability | North/South Central Coasts most vulnerable with high LVI-IPCC scores; exposed to floods, storms. Red River Delta, Northern Midlands, Central Highlands are moderately vulnerable. Red River and Mekong Deltas sensitive to food/water issues like drought and saltwater intrusion. |
| 105 | Vu | 2014 | Regional differences in the growing incidence of dengue Fever in Vietnam explained by weather variability. | 1999 | 2009 | Longitudinal/Time-series | Climate-sensitive infectious diseases | Multiple regions | Temperature, rainfall, humidity, sunshine hours | Dengue fever | Dengue cases rise 0.7% per 1mm increase in rainfall (0–3 month lag). Dengue cases increase significantly with 1°C rise in temperature; varies by location (Ha Noi highest at 135.1%). Positive association between 1% humidity increase and dengue cases in Khanh Hoa, Ho Chi Minh, Ca Mau. Negative association in Ha Noi with 1% humidity increase leads to 24.1% dengue decrease. Sunshine hours negatively associated with dengue in Ha Noi and Ca Mau, positively in Gia Lai. |
| 106 | Vu | 2017 | Flood risk assessment and coping capacity of floods in central Vietnam | 2012 | 2012 | Cross-sectional study | Extreme weather events & Vulnerability assessment | South Central Coast | Floods/rainfalls | vulnerability | In case of a 200-year return period flood: the estimated potential total damage in Quang Ngai province: 143,720 buildings damaged, 2910 hectares affected, number of injuries 630, number of deaths 64 In the case of a 100-year return period flood: the estimated damage in Quang Ngai province: total 137,080 buildings, 611 injuries, 63 deaths. |
| 107 | Wangdi | 2018 | Analysis of clinical malaria disease patterns and trends in Vietnam 2009-2015. | 2009 | 2015 | Longitudinal/Time-series | Climate-sensitive infectious diseases | Multiple regions | Temperature | malaria | P. falciparum to P. vivax ratio fell from 4.3 in 2009 to 0.8 in 2015. 1% ITN coverage increase led to 1.1% drop in P. falciparum incidence. 1°C temp increase led to 4% and 1.6% rise in P. falciparum and P. vivax, respectively. Higher than national average incidence in Central and Central-Southern districts. |
| 108 | Xuanle | 2014 | Seasonality in mortality and its relationship to temperature among the older population in Hanoi, Vietnam. | 2005 | 2010 | Longitudinal/Time-series | Temperature and health | Red River Delta | temperature | Mortality | High mortality peak in February 2008 due to low temperatures. Significant link between low temperature and mortality in older people. Death numbers dropped significantly in 2009. 21% more deaths in cold season vs. warm season. Higher cold-period mortality in females (IRR=1.23) than in males (IRR=1.18). |
| 109 | Xuanle | 2014 | Estimates of meteorological variability in association with dengue cases in a coastal city in northern Vietnam: an ecological study. | 2008 | 2012 | Longitudinal/Time-series | Climate-sensitive infectious diseases | Red River Delta | Temperature, (rainfall, humidity) | Dengue fever | Dengue risk linked to increased rainfall and humidity, RR 1.02-1.03 (p<0.05). Higher rainfall (RR 1.06) and humidity (RR 1.05) independently increase dengue risk. 11.7% of dengue cases attributed to climate factors. No association between elevated temperature and dengue occurrence. |
| 110 | Yamashita | 2022 | Effects of prenatal exposure to abnormal rainfall on cognitive development in Vietnam | 2002 | 2016 | Longitudinal/Time-series | Extreme weather events | Multiple regions | Floods/rainfalls | cognitive development | Above-average rainfall boosts cognitive development up to age 8. Stronger effects if positive shocks occur early in gestation. Effects are not found at ages 10, 12, and 15. |
| 111 | Zamand | 2016 | Impact of climatic shocks on child human capital: evidence from young lives data | 2009 | 2009 | Cross-sectional study | Extreme weather events | Multiple regions | drought and excessive rainfall/flood | children's health | Mean BFA and HFA z-scores are significantly below international standards. Droughts negatively impact child HFA z-scores, showing income effect dominates. |
| 1. **Vietnamese papers** | | | | | | | | | | | |
| 112 | Le H Ninh | 2016 | Tác Động Của Biến Đổi Khí Hậu Trên Bệnh Sốt Xuất Huyết Tại Khu Vực Nhà Bè Thành Phố Hồ Chí Minh Từ Năm 2000 Đến Năm 2014 (Impact of Climate Change on Dengue Fever in Nha Be Area of Ho Chi Minh City from 2000 to 2014) | 2000 | 2014 | Description study | Climate-sensitive infectious diseases | South East | seasonality | Dengue fever | The trend of dengue: increasing from 2000 and peaked from 2008-2011 then reduce till 2014. |
| 113 | Le H Ninh | 2016 | Liên Quan Bệnh Tay Chân Miệng Và Yếu Tố Khí Hậu Tại 6 Quận/ Huyện Thành Phố Hồ Chí Minh Giai Đoạn 2008 – 2014 (and, Foot and Mouth Disease and Climate Factors in 6 Districts of Ho Chi Minh City in the Period of 2008 - 2014) | 2008 | 2014 | Longitudinal/Time-series | Climate-sensitive infectious diseases | South East | Temperature | HFMD | Tmax increase of 1°C leads to significant rise in HFMD cases in specific districts. Climatic impact on HFMD varies by district. |
| 114 | Le H Ninh | 2016 | Biến Đổi Khí Hậu Và Ảnh Hưởng Lên Số Ca Mắc Cúm Tại Quận 5, Thành Phố Hồ Chí Minh (Climate Change and its Impact on the Number of Influenza Cases in District 5, Ho Chi Minh City) | 2006 | 2014 | Description study | Climate-sensitive infectious diseases | South East | Temperature | influenza-like illness | The average temperature in Ho Chi Minh City was at a high level, and then the number of influenza cases reduced when the temperature (max, average) increased. Influenza cases increase 16 cases with 1% increase in humidity |
| 115 | Le T Phung | 2016 | Nghiên cứu mối liên quan giữa sốt xuất huyết dengue và một số yếu tố vi khí hậu tại tỉnh Khánh Hòa trong 11 năm: 2004-2014 (the relationship between dengue hemorrhagic fever and some microclimatic factors in Khanh Hoa province for 11 years: 2004-2014) | 2004 | 2014 | Description study | Climate-sensitive infectious diseases | South Central Coast | Temperature, humidity, and precipitation | Dengue fever | Distribution of dengue cases were similar in Nha Trang and Cam Ranh; Postive association between dengue cases and temperature, humidity and rainfall; not significant with wind speed;  Model of three climate factors can explain 20% of dengue cases in Nha Trang and 30% cases in Cam Ranh |
| 116 | Nhu HV | 2014 | Đặc điểm tử vong và chấn thương do thảm họa tự nhiên tại Việt Nam từ năm 2000 đến năm 2012 (Characteristics of natural disaster-related death and injury in Viet Nam from 2000 to 2012) | 2000 | 2012 | Description study | Climate-sensitive infectious diseases | Multiple regions | Multiple extreme weather events | death, injuries | Natural disasters caused 4,765 deaths and 7,812 injuries from 2000-2012. Floods were the leading cause of death, followed by storms and flash floods. Mekong Delta had highest death rate; South Central region next. Majority of deaths were male (69.7%). Ages 15-24 and above 55 were most affected. Common death scenarios include sailing through floods and collecting assets in floodwater. |
| 117 | Thai Quang H | 2014 | Phân Bố Bệnh Tay Chân Miệng Và Mối Liên Quan Đến Một Số Yếu Tố Khí Hậu Ở Tỉnh Đắk Lắk Năm 2012-2013 (Hand-foot-mouth diseases and climate factors in Dak Lak province from 2012 to 2013)\ | 2012 | 2013 | Longitudinal/Time-series | Climate-sensitive infectious diseases | Central Highlands | Temperature, rainfall, humidity, sunshine hours | HFMD | Monthly: 1 degree C increase in temperature lead to 6.3% increase in HFMD cases.  1% increase in monthly humidity lead to 9.8% increase in HFMD cases. 50mm in rainfall is associated to increase by 1.7% HFMD 5 hour of sunlight is associated to increase by 3.4% HFMD Model of the 4 factors explained for 22% of increase in HFMD |
| 118 | Thao NT | 2021 | Nhu cầu đào tạo về biến đổi khí hậu và sức khỏe ở các Trường Đại học Y khoa tại Việt Nam (The need for training on climate change and health at Medical Universities in Vietnam) | 2018 | 2021 | survey | Awareness and Engagement | Multiple regions | Climate change in general | awareness | Medical students receive 1-2 lessons on climate change and health. Training is essential for both general and preventive medicine roles. Students want climate topics in 2nd-3rd year for general medicine, 4th-5th for preventive medicine. Preferred methods: presentations, reports, discussions. Topics should include health issues and response plans for climate change. |
| 119 | Nguyen Cong Trung D | 2020 | Mùa Truyền Bệnh Sốt Rét Trong Bối Cảnh Biến Đổi Khí Hậu Tại Phước Sơn (Bình Phước) Và Tà Cạ (Nghệ An) Năm 2013 - 2014 (Malaria tranmission season in the context of climate change in Phuoc Son (Binh Phuoc province) and Ta Ca (Nghe An province) in 2013-2014 | 2013 | 2014 | Description study | Climate-sensitive infectious diseases | Multiple regions | seasonality | malaria | Malaria peaks in Phuoc Son, Binh Phuoc from Sep-Nov, shorter than in Ta Ca, Nghe An, which is Jul-Nov. |
| 120 | Nguyen Phuong T | 2014 | Mối Liên Quan Giữa Sốt Xuất Huyết Và Biến Đổi Khí Hậu Tại Cần Thơ 2001-2011 (The Relationship Between Dengue Fever and Climate Change in Can Tho 2001-2011) | 2001 | 2011 | Description study | Climate-sensitive infectious diseases | Mekong River Delta | seasonality | Dengue fever | Dengue start to increase in April and peaked in October; the Effects of CC on dengue were not significant. 1-month lag will be the best for predict Dengue |
| 121 | Nguyen Thu H | 2015 | Sóng nhiệt và tình hình nhập viện của trẻ dưới 5 tuổi tại thành phố Vinh, tỉnh Nghệ An (Heat waves and hospitalization of children under 5 years old in Vinh city, Nghe An province) | 2010 | 2012 | Description study | Temperature and health | North Central Coast | seasonality | children's health | 2010 hottest year in three years, highest admission rates. June-Sep hotter than Feb-May, with more hospital admissions. Admissions for diarrhea and respiratory diseases higher in hot, dry months. 2010 had more hospitalizations for diarrhea and respiratory diseases compared to 2011 and 2012. |
| 122 | Nguyen Thi Lien H | 2016 | Dự báo tăng nhập viện liên quan tới nhiệt độ tại các bệnh viện tuyến trên thuộc khu vực Đồng bằng sông Cửu Long, Việt Nam (Forecasted increase in temperature-related hospitalizations at higher level hospitals in the Mekong Delta region, Vietnam) | 2000 | 2014 | Longitudinal/Time-series | Temperature and health & Future risks | Mekong River Delta | Temperature | Hospitalisation risk | Temperature is projected to increase in all provinces; 0.2-1.4 °C in 2030; --> 31.465 additional cases 0.3-12.5 °C in 2050; --> 36.171 additional cases 0.5-3.6 °C in 2070; --> 41.211 additional cases 0.8-4.7 °C in 2090; --> 46.582 additional cases 0.8-5.8 °C in 2100 --> 49.392 additional cases.  An Giang and Soc Trang will be affected the most in the MDR (10.000 additional cases in each province in 2100) |
| 123 | Nguyen Bich Diep | 2015 | Biến đổi khí hậu và Tai nạn thương tích cộng đồng tại một tỉnh ven biển, giai đoạn 2007-2011 (Climate change and community's injury abd dead in a coastal province during 2007-2011) | 2007 | 2011 | Description study | Extreme weather events | Red River Delta | Multiple extreme weather events | death, injuries | Injuries and deaths increase during stormy and very cold days. Traffic injuries are the leading cause, affecting mostly males and farmers. Most victims are of working age (15-60). Death rates rise with the increasing number of stormy and very cold days. |
| 124 | Nguyen Thi Minh N | 2015 | Kiến Thức Và Thực Hành Ứng Phó Với Biến Đổi Khí Hậu Của Người Dân Tại Xã Đông Ninh, Đông Sơn, Thanh Hóa Năm 2015 (Knowledge and Practice of Responding to Climate Change of People in Dong Ninh, Dong Son, Thanh Hoa Communes in 2015) | 2015 | 2015 | Incidence or prevalence study without comparison | Awareness and Engagement | North Central Coast | Climate change in general | awareness | 3.2% fully understand climate change causes. 26.7% have moderate awareness of climate change effects. 49.8% took action due to climate concerns. Overall, low proportion of people fully understand and act on climate change. |
| 125 | Nguyen Van H | 2015 | Kiến Thức, Thái Độ Về Biến Đổi Khí Hậu Và Chuẩn Bị Ứng Phó Với Biến Đổi Khí Hậu Của Người Dân 2 Xã Thuộc Huyện Bình Lục, Tỉnh Hà Nam Năm 2013 (Knowledge and Attitudes About Climate Change and Preparation to Respond to Climate Change of People in 2 Communes of Binh Luc District, Ha Nam Province in 2013) | 2013 | 2013 | survey | Awareness and Engagement | Red River Delta | Climate change in general | awareness | 41.3% unaware of climate change. 33.3% not interested in learning about it. Lack of preparation for climate-related diseases. High demand for climate change education and communication. |
| 126 | Mai Thi Cam V | 2018 | Nhận Thức Và Khả Năng Đáp Ứng Với Tác Động Của Biến Đổi Khí Hậu Đến Sức Khỏe Của Người Dân Xã Duy Tân, Huyện Duy Xuyên, Tỉnh Quảng Nam, Năm 2017 (Awareness and Ability to Respond to the Impact of Climate Change on the Health of People in Duy Tan Commune, Duy Xuyen District, Quang Nam Province, 2017) | 2017 | 2017 | Incidence or prevalence study without comparison | Awareness and Engagement | South Central Coast | Climate change in general | awareness | 89.02% aware of climate change. 74.35% believe it's human-caused. 79.87% think it affects health. Common adaptations: sun coat, warm baths, water storage, care for vulnerable. Education level not tied to climate perception. Need for support from medical staff and authorities for better adaptation. |
| 127 | Chinh VĐ | 2019 | Đánh Giá Tính An Toàn Của Cơ Sở Y Tế Tp. Hồ Chí Minh Trước Tác Động Của Biến Đổi Khí Hậu, Năm 2019 (Assessement of Hospital Safety in Ho Chi Minh City in Response to Climate Change in 2019) | 2018 | 2019 | Incidence or prevalence study without comparison | Adaptation and Policy | South East | Multiple extreme weather events | vulnerability | 22 hospitals studied: central, city, district, private. Most meet location and accessibility safety standards. Failures in design and structure: construction density (13.6%), fire safety (18.2%), earthquake and storm safety (18.2%). Non-structural safety issues: roof, ceiling, doors (4.5%-22.7%). Technical infrastructure issues: electricity, water, gas. 13.6% lack emergency and disaster information for patients and staff. |

# **Supplemental #4. Additional Analysis**

## **Heat Exposure Vulnerability Index (HEVI)**

HEVI = 100*(Pop65 + Major NCD Prevalence + Urbanization Rate)/3

- Pop65 is the proportion of the population aged 65 years or above. The data on the proportion of the Pop65 were obtained from the World Bank data (<https://data.worldbank.org/indicator/SP.POP.65UP.TO.ZS?locations=VN>)

- Major NCD Prevalence is the prevalence of cardiovascular, diabetes and chronic respiratory diseases among aged 65 years or above. Major Non-Communicable Disease prevalence is the prevalence of cardiovascular, diabetes and chronic respiratory diseases among the population aged 65 years or above. These data for 1990-2019 were also sourced from GBD 2019 (<http://ghdx.healthdata.org/gbd-results-tool>).

- Urbanization Rate is the proportion of people living in urban areas. The data on Urbanization rate was obtained from the World Bank data (<https://data.worldbank.org/indicator/SP.URB.TOTL.IN.ZS?locations=VN>)

## **Exposure of Vulnerable Population to Heatwaves (EVPH)**

The calculation of heatwaves in Vietnam utilizes data obtained from the ERA5 dataset. ERA5 stands for “the fifth generation of the European Centre for Medium-Range Weather Forecasts (ECMWF) Atmospheric Reanalysis of the Global Climate”. The ERA5 dataset offers a consistent and comprehensive view of the Earth's climate over the past 40 years, obtained through a combination of numerical models and observations from various sources, such as satellites, ground-based, and weather balloon measurements. This dataset comprises several variables, including temperature, wind, precipitation, and atmospheric pressure, at hourly intervals, and a grid resolution of approximately 31 km ^1^. Researchers in climate science, atmospheric physics, and environmental modelling have extensively employed ERA5 for various applications, including weather forecasting, climate analysis, and assessment of climate change impacts ^2^.

With the aim of capturing the health effects caused by both maximum and minimum temperatures, we adopted the heatwave definition reported in The Lancet countdown 2021 ^3^. More specifically, each heatwave is identified when both the daily maximum and minimum temperatures simultaneously exceed the 95^th^ percentile of their respective climatology for a period of at least two consecutive days. The climatology used here is 30 years from 1991 to 2020. The heatwaves calculated in this manner, thus, allow accounting for the combined effects of both direct heat extremes caused by high maximum temperatures and sustained hot periods without proper recovery caused by high minimum temperatures. This definition is also in line with the World Meteorological Organization and published scholarly literature ^3^.

The exposure of vulnerable populations to heatwaves (VPH) is quantified in person-days for each year as the number of people exposed to the number of heatwaves, i.e., EVPH = heatwave days x people affected.

**“Back-of-the-envelop” approach**

## “Back-of-the-envelop” approach to estimate the costs of climate change on human health

To estimate the costs of climate change on human health, we combine marginal effects parameters, projections of climate change impacts, GDP, and the population of Vietnam from various studies.

A major effect of climate change is raising temperature. The World Bank estimated that the average temperature in Vietnam would increase by 1-3·4^o^C as a result of climate change.^4^ Using Nguyen 2021’s marginal effects (i.e., a 1^0^C increase in average monthly temperature is associated with a 5% increase in health expenditure),^5^ and data from the World Bank on Vietnam’s health expenditure (5·3% of GDP, and Vietnam’s GDP projection by 2050 is $500 billion),^6,7^ we estimated that the healthcare costs associated with raising temperature due to climate change range from $2·65 billion to $9·0 billion. Using a discount rate of 5%, the healthcare costs of rising temperature due to climate change at present value is $0·6 billion-$2·0 billion. This figure may be a lower bound because it is expected that by 2050 Vietnam will face the population aging issue, which will result in higher health expenditure. Assuming that by 2050, Vietnam will increase health expenditure to 9% of GDP, which is the average figure among the Organisation for Economic Co-operation and Development (OECD) countries at present, the effects of climate change on health expenditure in Vietnam will range from $1·0 billion-$3·4 billion at present value.

Apart from illness, climate changes also result in increased fatalities. According to an estimate by the World Bank, climate change is expected to cause 125 deaths per one million population due to food shortages.^4^ Using Vietnam’s population projection of 120 million by 2050,^8^ and the estimated value of statistical life (VLS) in Vietnam ranged from Vietnam Dong (VND) 4·4 billion ($200,000) when using willingness-to-pay (WTP) to VND 29·5 billion ($1·3 million)^9^ when using the willingness-to-accept (WTA) for the risk of cancer, the cost of climate change effects on premature deaths ranged from $3 billion to $20 billion. Heat-related illness also incurs costs of productivity loss due to work absentees. The estimated work productivity loss due to heat ranges from 5% to 20% of GDP by 2050 (figure above), which translate to $25 billion to $100 billion given the projected GDP of Vietnam is $500 billion by 2050.^7^ Using a discount rate of 5%, the cost of work capacity loss due to climate changes varies from $6 billion to $23 billion.

Overall, the disease burden of climate change in Vietnam is substantial at $1b-$3bn per year of healthcare costs and $3b-$20b associated with premature deaths. The largest cost component, however, is the loss of work capacity at $6b to $23b by 2050.

## References

1. Hersbach H, Bell B, Berrisford P, Hirahara S, Horányi A, Muñoz‐Sabater J, et al. The ERA5 global reanalysis. Quarterly Journal of the Royal Meteorological Society. 2020;146(730):1999-2049.

2. Urban A, Di Napoli C, Cloke HL, Kyselý J, Pappenberger F, Sera F, et al. Evaluation of the ERA5 reanalysis-based Universal Thermal Climate Index on mortality data in Europe. Environmental research. 2021;198:111227.

3. Romanello M, McGushin A, Di Napoli C, Drummond P, Hughes N, Jamart L, et al. The 2021 report of the Lancet Countdown on health and climate change: code red for a healthy future. The Lancet. 2021;398(10311):1619-62.

4. World Bank, Asian Development Bank. Climate Risk Country Profile: Vietnam: World Bank; 2021.

5. Nguyen CV. Do weather extremes induce people to move? Evidence from Vietnam. ECONOMIC ANALYSIS AND POLICY. 2021;69:118-41.

6. Current health expenditure (% of GDP) - Vietnam [Internet]. 2023. Available from: <https://data.worldbank.org/indicator/SH.XPD.CHEX.GD.ZS?locations=VN>.

7. Arndt C, Tarp F, Thurlow J. The Economic Costs of Climate Change: A Multi-Sector Impact Assessment for Vietnam. 2015;7(4):4131-45.

8. World Bank. Vietnam Country - Overview 2023 [cited 2023 April 05, 2023]. Available from: <https://www.worldbank.org/en/country/vietnam/overview>.

9. Kiet NT, Knetsch J. Is the Value of Statistical Life Sensitive to the Choice of Measure?: Evidence from Transportation Accident and Cancer Risks in Vietnam. 2021.

# **Supplemental #5. Keywords (Vietnamese) used for searching media.**

"biến đổi khí hậu", “nóng ấm toàn cầu”, “nóng lên toàn cầu”, “hiệu ứng nhà kính”, "phát thải các bon", “thảm hoạ thiên nhiên”, “hiện tượng thời tiết khắc nghiệt”, “dao động thời tiết”, “sóng nhiệt”, “lạnh cực đoan”, “mưa cực đoan”, “hạn hán”, “rét đậm rét hại”, “lũ lụt", “thiên tai”, “các yếu tố liên quan đến khí hậu”, "Sức khoẻ con người", “sức khoẻ”, “bệnh tật”, “sức khoẻ cộng đồng”, “gánh nặng bệnh tật”, “bệnh tật”, “tử vong”, “chấn thương”, “bệnh truyền qua véc tơ”, "bệnh do muỗi truyền", “bệnh nhạy cảm với khí hậu”, “sức khoẻ tâm thần”.

# **Supplemental #6. List of key policies and strategies for climate change adaptation in Vietnam (1994-2022)**


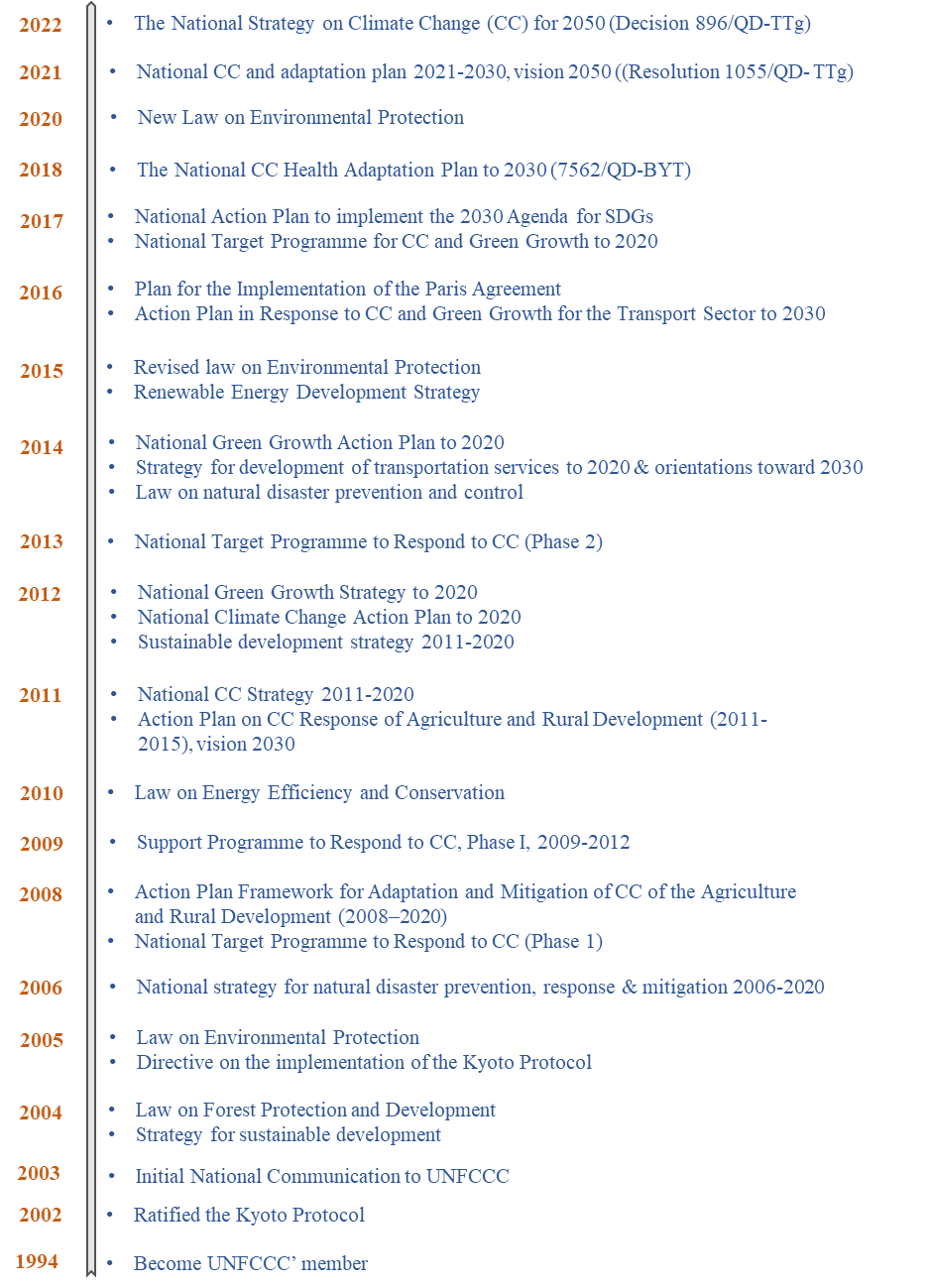

Supplement: Supplement [file mmc1.docx]
