# Supplementary material for: Climate change and human health in Vietnam: a systematic review and additional analyses on current impacts, future risk, and adaptation
Source: Lancet Reg Health West Pac. 2023 Nov 15;40:100943. doi: 10.1016/j.lanwpc.2023.100943 (PMC10730327; doi:10.1016/j.lanwpc.2023.100943)
Supplement: Translated Abstract [file mmc2.docx]

**Tóm tắt**

Nghiên cứu này nhằm mục đích đánh giá tác động của biến đổi khí hậu (BĐKH) đến sức khỏe và khả năng thích ứng ở Việt Nam thông qua xem xét tổng quan có hệ thống và tiến hành các phân tích bổ sung về nguy có phơi nhiễm với nhiệt độ cao, tính dễ bị tổn thương do nhiệt, nhận thức về BĐKH và sự tham gia trong thích ứng với BĐKH cũng như ước tính chi phí y tế liên quan đến BĐKH.

Trong số 127 nghiên cứu được xem xét, các phát hiện cho thấy sự lây lan rộng hơn của các bệnh truyền nhiễm, đồng thời tăng nguy cơ tử vong và nhập viện liên quan đến nắng nóng khắc nghiệt, hạn hán và lũ lụt. Tuy nhiên, có rất ít nghiên cứu đề cập đến chi phí y tế, nhận thức, sự tham gia, khả năng thích ứng và chính sách.

Các phân tích bổ sung cho thấy mức độ phơi nhiễm với sóng nhiệt ngày càng gia tăng trên khắp Việt Nam và mức độ dễ bị tổn thương trước nhiệt độ trên mức trung bình toàn cầu. Đến năm 2050, dự kiến biến đổi khí hậu sẽ gây thiệt hại lên tới 1-3 tỷ USD cho chi phí chăm sóc sức khỏe, 3-20 tỷ USD cho trường hợp tử vong sớm và 6-23 tỷ USD cho tình trạng mất việc làm.

Mặc dù các phương tiện truyền thông ngày càng tập trung vào khí hậu và sức khỏe, các bản tin đến từ công chúng nhiều hơn từ cơ quan chính phủ làm nổi bật sự cần thiết phải có sự tham gia nhiều hơn của chính phủ. Các chính sách thích ứng với BĐKH của Việt Nam phải đối mặt với những thách thức khi thực hiện, bao gồm cách tiếp cận từ trên xuống, thiếu hợp tác giữa các ban ngành, năng lực thích ứng với BĐKH thấp và nguồn lực hạn chế.
